# Quantum Chemical Characterization of Rotamerism in Thio-Michael Additions for Targeted Covalent Inhibitors

**DOI:** 10.1021/acs.jcim.4c01379

**Published:** 2024-09-12

**Authors:** Shayantan Chaudhuri, David M. Rogers, Christopher J. Hayes, Katherine Inzani, Jonathan D. Hirst

**Affiliations:** School of Chemistry, University of Nottingham, Nottingham NG7 2RD, U.K.

## Abstract

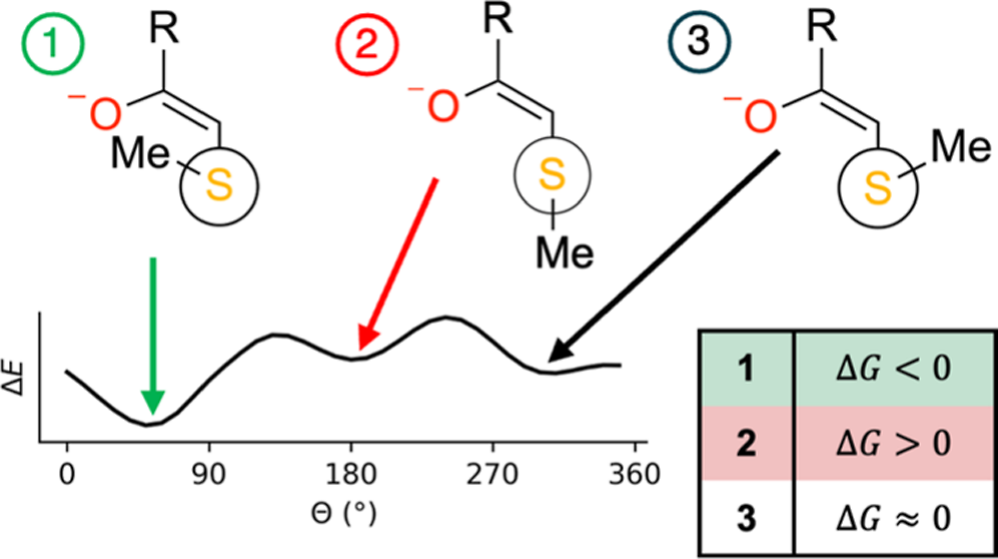

Myotonic dystrophy type I (DM1) is the most common form of adult muscular dystrophy and is a severe condition with no treatment currently available. Recently, small-molecule ligands have been developed as targeted covalent inhibitors that have some selectivity for and covalently inhibit cyclin-dependent kinase 12 (CDK12). CDK12 is involved in the transcription of elongated RNA sections that results in the DM1 condition. The covalent bond is achieved after nucleophilic addition to a Michael acceptor warhead. Previous studies of the conformational preferences of thio-Michael additions have focused on characterizing the reaction profile based on the distance between the sulfur and β-carbon atoms. Rotamerism, however, has not been investigated extensively. Here, we use high-level quantum chemistry calculations, up to coupled cluster with single, double, and perturbative triple excitations [CCSD(T)], to characterize the nucleophilic addition of an archetypal nucleophile, methanethiolate, to various nitrogen-containing Michael acceptors which are representative of the small-molecule covalent inhibitors. By investigating the structural, energetic, and electronic properties of the resulting enolates, as well as their reaction profiles, we show that synclinal additions are generally energetically favored over other additions due to the greater magnitude of attractive noncovalent interactions permitted by the conformation. The calculated transition states associated with the addition process indicate that synclinal addition proceeds via lower energetic barriers than antiperiplanar addition and is the preferred reaction pathway.

## Introduction

Myotonic dystrophy type I (DM1) is a severe, progressive, and debilitating condition which affects a range of human physiological systems, including skeletal, muscular, cardiac, and neural. DM1 is the most common form of adult muscular dystrophy with a global incidence of over 1 in 8000 people,^1^ and there is no treatment currently available. The genetic basis of DM1 is a CTG trinucleotide repeat sequence in the 3′ untranslated region of the dystrophia myotonica protein kinase gene.^2−4^ While the faulty DNA is correctly transcribed into RNA and spliced, it is too large to leave the nucleus and instead gets trapped there, sequestering muscleblind-like and other cell proteins to form distinct microscopically detectable nuclear foci.^5−7^ Being an RNA disorder, DM1 lacks a clear protein target for drug development; however, cyclin-dependent kinase 12 (CDK12) has recently been identified as a potential target.^8^ CDK12 is a ∼164 kDa protein comprising 1490 amino acids that is located in human chromosome 17q12 and is composed of 14 exons.^9^ CDK12 is involved in the transcription of elongated RNA sections, resulting in mutant CUG RNA repeats. The inhibition of CDK12 reduces the transcription of faulty RNA and therefore could potentially treat DM1.^8^ Furthermore, the development of CDK12 inhibitors could be used to treat other disorders^10,11^ and even some cancers.^12,13^

**Figure 1:**
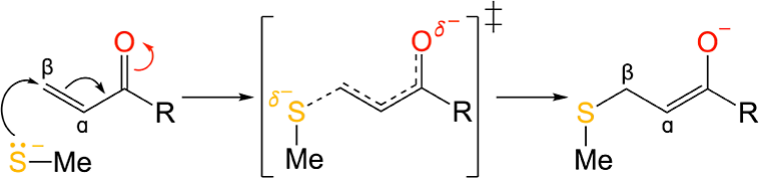
General mechanism of the nucleophilic addition of methanethiolate to a Michael acceptor, forming an anionic enolate intermediate via a transition state (labeled as ‡). The terminal group of the Michael acceptor is denoted as R, and both the α- and β-carbon atoms are also labeled. The methyl (CH_3_) group is denoted as Me.

Novel small-molecule CDK12 inhibitors^14^ remove nuclear foci and improve myotonia in patient-derived cell lines and a mouse model.^8^ Selectivity for CDK12 over other proteins, such as CDK9, is achieved via the formation of a covalent bond with the cysteine 1039 residue within CDK12. The covalent bond is formed after a nucleophilic addition to a Michael acceptor warhead. In general, nucleophilic additions of thiols to Michael acceptors are key bond-forming processes in the burgeoning field of covalent drug design.^15−19^ The addition of thiols to Michael acceptors, which are α, β-unsaturated carbonyls and their derivatives, is typically considered to involve three key steps.^20^ First, the thiol is deprotonated to form a reactive thiolate anion.^21−23^ The nucleophilic thiolate then attacks the β-carbon atom (C^β^) of the Michael acceptor, giving rise to an anionic enolate intermediate, as shown in Figure 1. The third step is the protonation of the enolate, which results in the formation of a neutral adduct. The second step in this process is the rate-determining step, and it is therefore crucial to understand the reaction pathways associated with this step.

The nucleophilic addition of thiolates to Michael acceptors have been studied previously using computational methods.^17,24−29^ Studies typically aim to characterize anionic transition states^24,26^ or compare different electronic structure methods to determine the most appropriate method to model biochemical thio-Michael additions.^25,27^ For example, by calculating reaction profiles based on the S–C^β^ distance, Smith et al.^25^ showed that range-separated hybrid density-functional approximations were necessary to predict stable gas-phase enolates, as other common functionals suffer from spurious delocalization errors that stabilize noncovalently bound charge–transfer complexes. While valuable insights into the reaction mechanism can be elucidated from distance-dependent reaction profiles, such as the bond forming and breaking processes, a fuller understanding of the overall reaction profile requires consideration of molecular conformations. Rotamerism can play a key component on the steric effects, electronic effects, and selectivities exhibited by the products of thio-Michael additions. Furthermore, different rotamers can possess different reaction pathways and therefore exhibit alternative transition states and intermediates. It is thus crucial to characterize rotamerism in thio-Michael additions on an atomistic level. In the gas phase, synclinal addition has been observed to be more favorable than anticlinal due to charge–charge interactions,^28,29^ but these interactions are yet to be fully characterized, and rotamerism is still to be explicitly studied.

In this paper, we use quantum chemical methods to characterize the nucleophilic addition of methanethiolate, which is typically used as the archetypal nucleophile due to its convenience,^24,25,27,29^ to various nitrogen-containing Michael acceptor molecules which are representative of the small-molecule CDK12 inhibitors. We investigate the structural and energetic properties of various enolate rotamers and investigate their electronic properties to determine the most favorable conformations. We also characterize transition states and reaction pathways for enolate formation to understand the associated reaction profiles. In addition to gas-phase calculations, the influence of implicit solvent environments on the conformational energetics is investigated using models for protein and water.

## Methods

**Figure 2:**
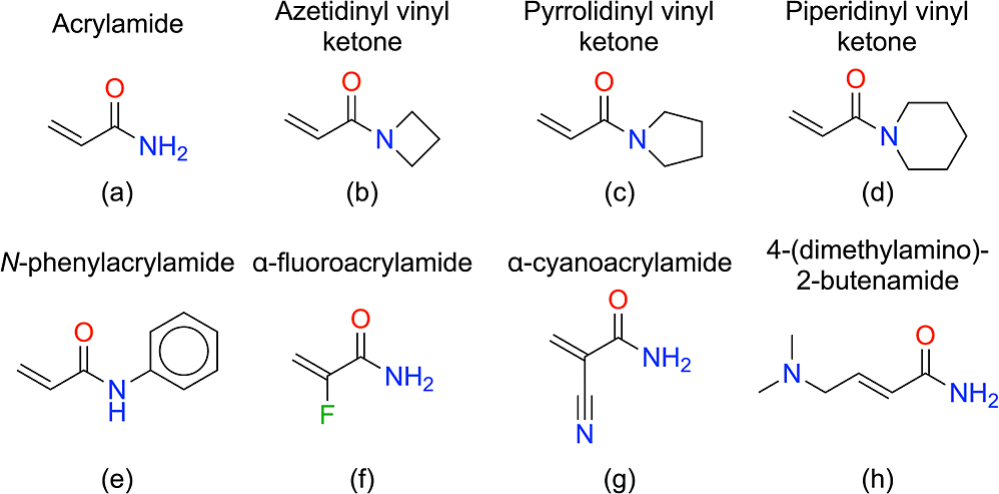
Chemical structures of the Michael acceptor warhead molecules studied herein. Shown are (a) acrylamide, (b) azetidinyl vinyl ketone, (c) pyrrolidinyl vinyl ketone, (d) piperidinyl vinyl ketone, (e) *N*-phenylacrylamide, (f) α-fluoroacrylamide, (g) α-cyanoacrylamide, and (h) 4-(dimethylamino)-2-butenamide.

The following warhead molecules were investigated: acrylamide, azetidinyl vinyl ketone, pyrrolidinyl vinyl ketone, piperidinyl vinyl ketone, *N*-phenylacrylamide, α-fluoroacrylamide, α-cyanoacrylamide, and 4-(dimethylamino)-2-butenamide, which are shown in Figure 2. These examples cover both reversible and irreversible inhibitors and allow us to examine a range of electronic and steric influences. The ASE^30^ and Jmol^31^ software packages were used to create and visualize molecular structures.

### Quantum Chemical Calculations

Quantum chemical calculations were performed using version 5.0.4 of the ORCA software package.^32,33^ Kohn–Sham density functional theory (DFT)^34,35^ calculations were performed using the ωB97X-D3(BJ)^36^ density-functional approximation, where ωB97X is the range-separated hybrid generalized gradient approximation^37^ and D3(BJ) is the DFT-D3^38^ dispersion correction with Becke–Johnson damping,^39^ which was included to account for long-range van der Waals interactions. ωB97X-D has previously been shown to be able to reproduce geometries and energetics calculated using ab initio wave function methods, unlike other common density-functional approximations which do not predict stable gas-phase enolates.^25,27^ Some wave function calculations were also performed, where geometries were optimized using second-order Møller–Plesset perturbation theory (MP2),^40^ and subsequent coupled cluster with single, double, and perturbative triple excitations [CCSD(T)]^41^ calculations were performed on the MP2-optimized geometries; this method is henceforth denoted as CCSD(T)@MP2.

All calculations were performed using the augmented correlation-consistent triple-ζ Dunning (aug-cc-pVTZ)^42,43^ basis set. Auxiliary basis sets supporting the resolution-of-identity (RI) approximation were also employed to reduce computational costs. For DFT calculations, the chain-of-spheres algorithmic approximation (RIJCOSX)^44^ for Coulomb and Hartree–Fock exchange matrix computations was used, with the general Weigend JK (aug-cc-pVTZ/JK)^45^ auxiliary basis set being used alongside aug-cc-pVTZ. For MP2 calculations, the RI approximation for MP2 integrals^46^ was used, and the Hellweg (aug-cc-pVTZ/C)^47^ auxiliary basis set was used to support MP2 calculations. The RIJCOSX^44^ approximation for the Hartree–Fock step within MP2 was also employed using aug-cc-pVTZ/JK.

In all calculations, the charge was set to −1 *e* and 0 *e* for anionic and neutral structures, respectively. All structures were close-shelled with no unpaired electrons, and the spin multiplicity was thus set to 1 in all calculations to represent the singlet state. Relaxed potential energy surface (PES) scans were conducted for both the S–C^β^ bond length, *r*, and the C^α^–C^β^–S–C dihedral angle, Θ. For *r*, the reaction coordinate was varied from 1.8 to 2.7 Å with 0.025 Å intervals, and the rest of the system allowed to relax; the S–C^β^ bond lengths of enolates and transition states are denoted as *r*_min_ and *r*^‡^, respectively. For Θ, the reaction coordinate was varied from 0 to 350° with 10° intervals, and the rest of the system was allowed to relax; the C^α^–C^β^–S–C dihedral angles of enolates and transition states are denoted as Θ_min_ and Θ^‡^, respectively.

Relative energies, Δ*E*, were calculated as the difference between the energy of the combined structure and the sum of the energies of the isolated reactants. Relative energies of enolates and transition states, Δ*E*_min_ and Δ*E*^‡^, respectively, were calculated as the difference between the energy combined structure and the sum of the energies of the isolated reactants. Harmonic vibrational frequencies were calculated numerically to confirm the nature of stationary points along the PES and to calculate Gibbs free energies.^48,49^ Relative Gibbs free energies of enolates and transition states, Δ*G*_min_ and Δ*G*^‡^, respectively, were calculated as the difference between the Gibbs free energy of the combined structure and the sum of the Gibbs free energies of the isolated reactants. Torsional energetic barriers, Δ*G*_Θ↑_^‡^ and Δ*G*_Θ↓_^‡^, between two enolates with dihedral angles 0° ≤ Θ_1_ < Θ_2_ < 360° were calculated as the difference between the Gibbs free energy of the torsional transition state and the Gibbs free energy of the enolate with Θ = Θ_1_ and Θ = Θ_2_, respectively. Enolates were obtained after reoptimization of energetic minima, without any geometry constraints, and their nature was confirmed by virtue of possessing all real frequencies. To obtain transition states, energetic maxima were reoptimized to a first-order saddle point, without any geometry constraints, and their nature was confirmed with the presence of one imaginary frequency.

A selection of calculations were also performed using implicit solvent environments; this was achieved by using the conductor-like polarizable continuum^50^ model within ORCA. A dielectric constant of 4 was used to model an implicit protein environment,^51^ as is commonly used to account for electronic polarization and small backbone fluctuations.^52^ A dielectric constant of 80.4 (at 25 °C) and a refractive index of 1.33 was used to model an implicit water environment.^51^ For any given structure, its Gibbs free energy of solvation, Δ*G*_solv_, was calculated as the Gibbs free energy in each-implicit solvent minus the Gibbs free energy in the gas phase i.e. a more negative Δ*G*_solv_ indicates greater stabilization by the solvent.

### Non-covalent Interactions

The NCIPLOT^53^ program was used to calculate intramolecular interactions.^54^ This was done by using the electron density, ρ, from ORCA calculations to calculate the reduced density gradient, *s*(ρ), which is a scalar field of ρ that describes the deviation from a homogeneous electron distribution,^34,55^ and is defined in eq 1
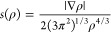
1where ∇ρ is the first derivative of ρ. Here, we investigated the relationship between *s*(ρ) and sgn(λ_2_)ρ to understand noncovalent interactions, where λ_2_ is the second eigenvalue of the Hessian matrix of the electron density.^53,54^ While ρ itself provides information regarding the strength of interactions, sgn(λ_2_) can be used to distinguish between bonded (λ_2_ < 0) and nonbonded (λ_2_ > 0) interactions,^53,54^ which is why sgn(λ_2_)ρ was used *in lieu* of ρ.

To create noncovalent interaction isosurfaces, the electron density from ORCA was used to write a Gaussian cube file with a grid interval resolution of 120 × 120 × 120. The following isosurface parameters were used: a surface cutoff of *s*(ρ) = 0.5 au and density cutoffs of 0.0005 au < ρ < 0.03 au The maximal cutoff was chosen to be ρ < 0.03 au as it encapsulates the noncovalent interaction regions of interest and removes covalent density, while ρ > 0.0005 au was chosen as the minimal cutoff to remove very low density points.^54^

## Results and Discussion

### Reaction Profiles and Transition States

**Figure 3:**
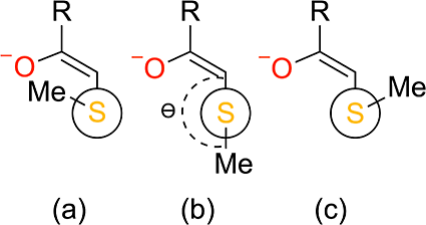
Newman projections of the three trajectories considered for nucleophilic addition between methanethiolate and different warheads. Shown are (a) synclinal, (b) antiperiplanar, and (c) synclinal. Also shown in (b) is the C^α^–C^β^–S–C dihedral angle, Θ, itself. The projection is shown from the viewpoint of the S–C^β^ bond and the terminal group of the warhead is denoted as R. The methyl (CH_3_) group is denoted as Me.

Three appropriate surfaces were considered for nucleophilic addition: two synclinal and one antiperiplanar, as visualized in the Newman projections^56^ in Figure 3. While synclinal versus antiperiplanar addition have been studied to a limited extent, studies do not consider the second synclinal conformation shown in Figure 3c.^17,26,28,29^

**Figure 4:**
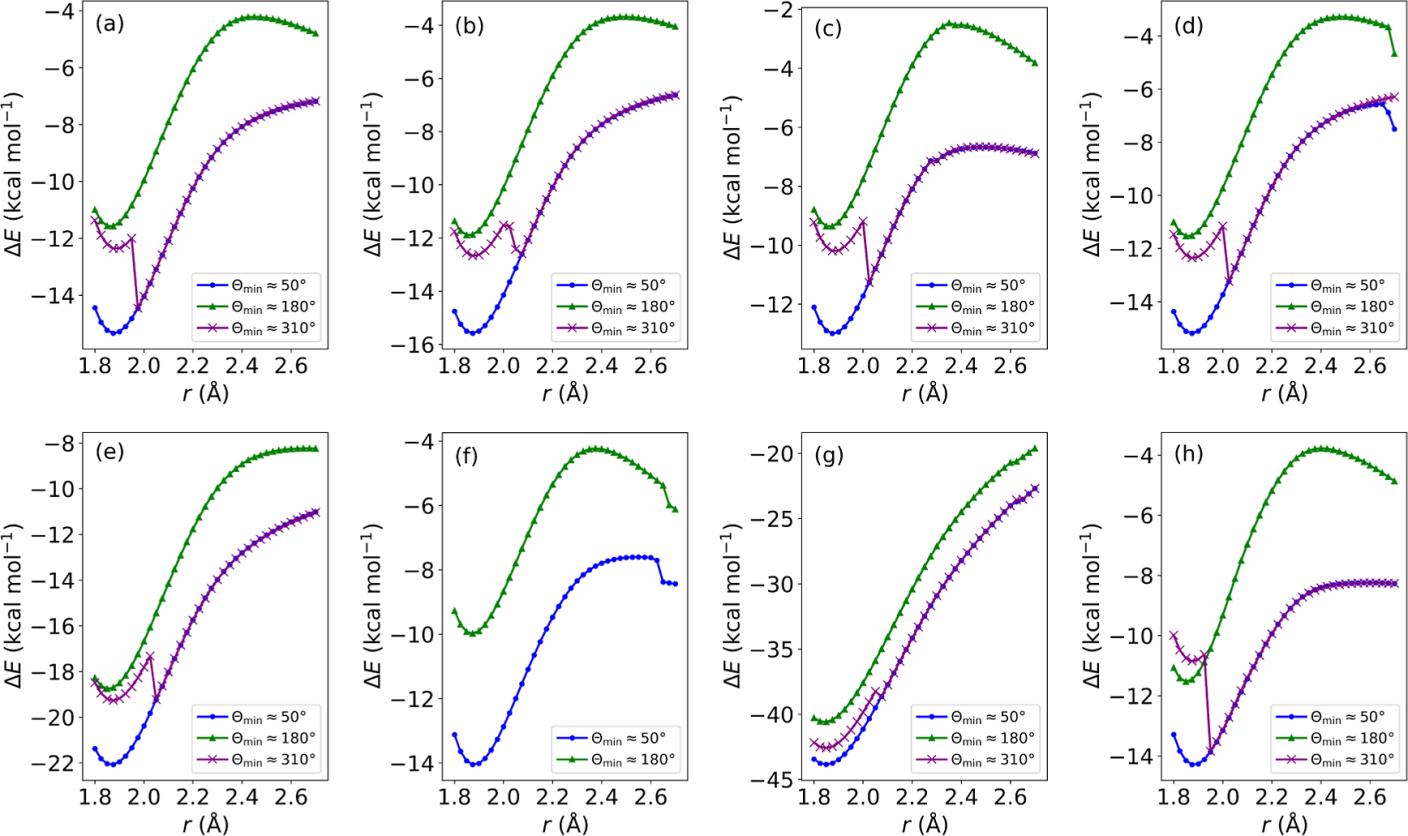
PES scans showing the relative energy, Δ*E*, with respect to the energies of the reactants for the S–C^β^ bond length, *r*, during the nucleophilic addition of methanethiolate to (a) acrylamide, (b) azetidinyl vinyl ketone, (c) pyrrolidinyl vinyl ketone, (d) piperidinyl vinyl ketone, (e) *N*-phenylacrylamide, (f) α-fluoroacrylamide, (g) α-cyanoacrylamide, and (h) 4-(dimethylamino)-2-butenamide. All values calculated at the ωB97X-D3(BJ)/aug-cc-pVTZ level.

PES scans, with *r* as the reaction coordinate, were performed to acquire reaction profiles for the three surfaces (see Figure 4). We note that for α-fluoroacrylamide, we were unable to plot a reaction profile for the second-synclinal surface (Θ ≈ 310°), as depicted in Figure 3c (*vide infra*). As shown in Figure S1(a,b), the PES scans calculated with CCSD(T)@MP2 and ωB97X-D3(BJ) closely match each other, with a mean absolute error of 1.15 kcal mol^–1^ between the Δ*E* values calculated with ωB97X-D3(BJ) and CCSD(T)@MP2. The CCSD(T) calculations were found to have a mean average *T*_1_ diagnostic value, which is the Euclidean norm of the singles amplitudes vector of the CCSD(T) wave function normalized by the square root of the number of correlated electrons,^57^ of 0.016 and a standard deviation of 0.001. The dependence of *T*_1_ on *r* can be seen in Figure S4(a,b). As the *T*_1_ values are less than 0.02, the CCSD(T) results are reliable,^58^ and the energetics calculated with ωB97X-D3(BJ) can thus be deemed to be accurate. We also note that Δ*E* does not converge to 0 kcal mol^–1^ as *r* → 2.7 Å. Similar trends have been reported for gas-phase thio-Michael additions due to the existence of a second energetic minimum at larger *r*, which corresponds to a contact pair and occurs due to a strong attractive force between the polar warhead and the anionic methanethiolate.^27^ Furthermore, Figures S2 and S3 show that the Mulliken charge on the sulfur atom tends toward −1 *e* for *r* > *r*_min_, confirming the S–C^β^ bond fission process to be heterolytic and that the negative charge is localized on the sulfur atom as *r* increases, as expected.

For all warheads, there is an energetic minimum at *r* ≈ 1.85 Å for all the PES scans in Figure 4, with the same stability trend observed between the surfaces. Across all warheads, the PES scans corresponding to the first-synclinal (Θ ≈ 50°) and antiperiplanar (Θ ≈ 180°) surfaces are continuous, but there is a point of discontinuity at *r* ≈ 2.0 °A for each of the PES scans corresponding to the second-synclinal surfaces (Θ ≈ 310°). This occurs because at around *r* > 2.0 Å, there is a noticeable change in the C^α^–C^β^–S–C dihedral angle in the *r*-constrained optimizations. From this point, the structures along the PES scan corresponding to the second-synclinal surface follow that of the first-synclinal surface, and this can be seen by the overlapping PES scans corresponding to these two surfaces after the point of discontinuity for all eight warheads in Figure 4. In fact, the coefficient of determination (*R*^2^) values between the overlapping regions of the synclinal PES scans were calculated to be 1.0 for each warhead, showing the high degree of correlation between the two regions.

The energetic minima for each warhead from Figure 4 were reoptimized (without any geometry constraints) to obtain their respective enolates, and thermal corrections to the electronic energies were calculated in order to determine relative Gibbs free energies. The enolates considered herein are also the most energetically stable as compared to their *E*-stereoisomers and other conformations based on pyramidalization at the nitrogen, as can be seen in Table S1 and visualized in Figure S5. Energetic maxima in the PES scans were reoptimized to a first-order saddle point to obtain transition states and reaction barriers. The structural and energetic characteristics of enolates and transition states are summarized in Table 1.

**Table 1:** Structural and Energetic Characteristics of the Enolates and Transition States after the Nucleophilic Addition of Methanethiolate to Various Warheads in Three Different Conformations^a^

warhead	Θ_min_ (deg)	*r*_min_ (Å)	Δ*E*_min_	Δ*G*_min_	Θ^‡^ (deg)	*r*^‡^ (Å)	(cm^–1^)	Δ*E*^‡^	Δ*G*^‡^
acrylamide	51.7	1.88	–15.34	–2.48	13.2	2.70	27.2	–8.31	+3.85
	182.0	1.86	–11.59	+1.01	182.1	2.44	137.6	–4.21	+7.18
	309.0	1.89	–12.37	–0.09					
azetidinyl vinyl ketone	51.7	1.88	–15.58	–2.82					
	183.9	1.86	–11.90	+0.25	177.9	2.50	106.2	–3.69	+7.65
	305.6	1.88	–12.67	–0.39					
pyrrolidinyl vinyl ketone	51.3	1.88	–13.00	–0.65	16.2	2.51	82.0	–6.66	+4.66
	183.2	1.86	–9.39	+2.20	178.8	2.39	182.9	–2.53	+8.36
	307.6	1.89	–10.20	+1.80					
piperidinyl vinyl ketone	52.9	1.87	–15.19	–2.21	18.1	2.54	85.3	–7.41	+4.65
	182.7	1.86	–11.55	+0.77	179.4	2.49	111.7	–3.27	+7.92
	306.9	1.88	–12.36	+0.10					
*N*-phenylacrylamide	54.3	1.87	–22.10	–9.58	18.1	3.15	66.2	–9.73	+0.86
	181.3	1.85	–18.77	–6.89	179.5	2.68	55.5	–8.24	+2.43
	304.7	1.87	–19.28	–7.53					
α-fluoro-acrylamide	51.4	1.89	–14.05	–1.48	16.6	2.55	92.4	–7.60	+3.86
	183.2	1.87	–9.98	+1.85	176.6	2.38	192.4	–4.23	+6.71
α-cyano-acrylamide	51.0	1.85	–43.86	–30.69					
	177.9	1.84	–40.59	–27.61					
	303.2	1.85	–42.58	–29.22					
4-(dimethylamino)-2-butenamide	50.6	1.88	–14.30	–0.35	18.3	2.58	56.7	–8.26	+4.81
	168.5	1.85	–11.53	+2.42	148.2	2.40	183.9	–3.78	+8.58
	307.6	1.88	–10.85	+2.34					

a The following are included for the enolates: the C^α^–C^β^–S–C dihedral angles (Θ_min_), S–C^β^ bond lengths (*r*_min_), relative energies (Δ*E*_min_), and relative Gibbs free energies (Δ*G*_min_). The following are included for the transition states: the C^α^–C^β^–S–C dihedral angle (Θ^‡^), S–C^β^ bond lengths (*r*^‡^), magnitude of the frequencies of the sole imaginary mode , relative energies (Δ*E*^‡^), and relative Gibbs free energies (Δ*G*^‡^). Note that no transition states were obtained for the Θ_min_ ≈ 310° minima; see main text for details. All values were calculated at the ωB97X-D3(BJ)/aug-cc-pVTZ level and all energies (Δ*E*_min_, Δ*G*_min_, Δ*E*^‡^, and Δ*G*^‡^) are in kilocalories per mole (kcal mol^–1^).

**Figure 5:**
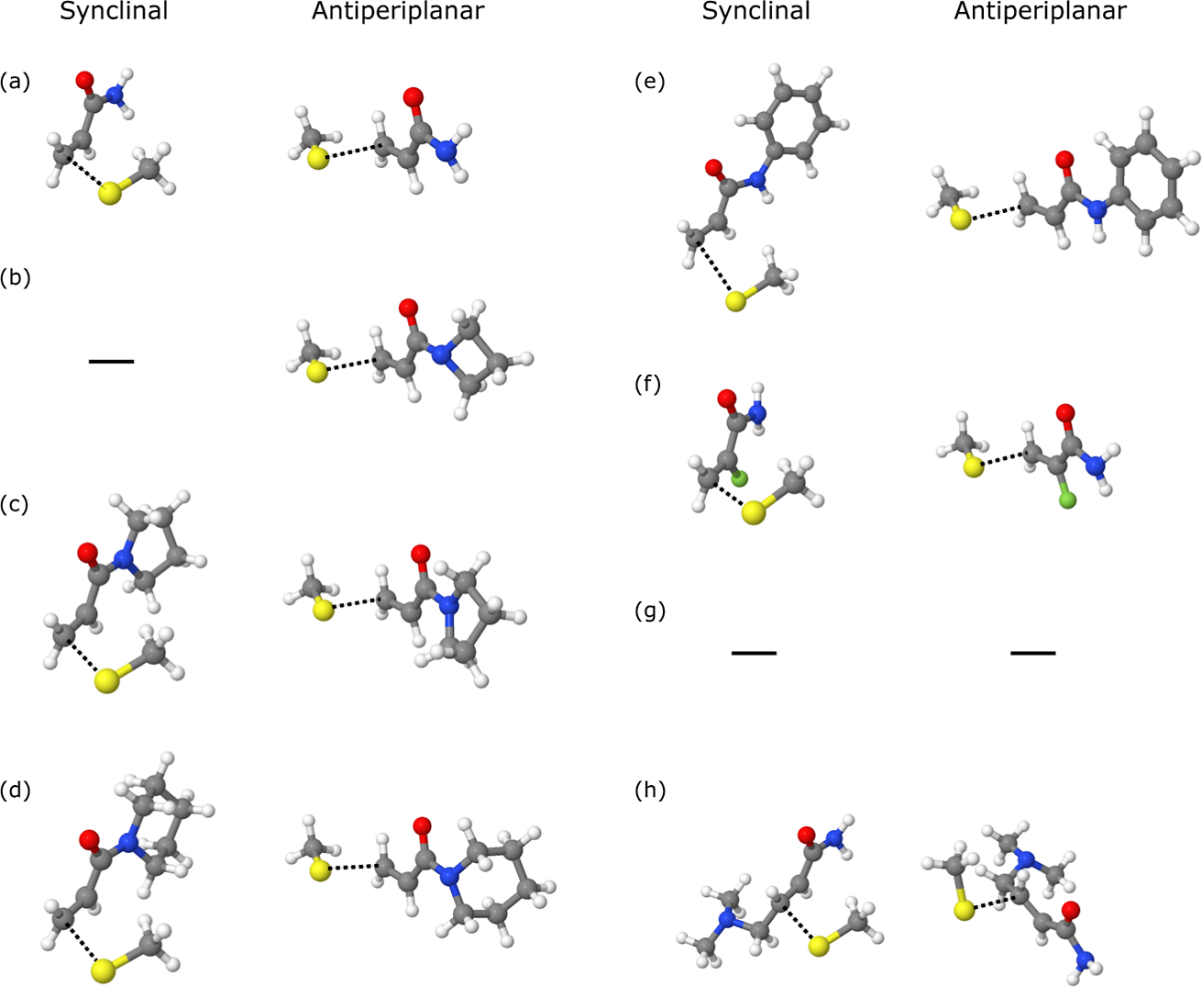
Visualizations of transition states during the nucleophilic addition of methanethiolate to various Michael acceptor warheads. Shown are synclinal (first column) and antiperiplanar (second column) additions to (a) acrylamide, (b) azetidinyl vinyl ketone, (c) pyrrolidinyl vinyl ketone, (d) piperidinyl vinyl ketone, (e) *N*-phenylacrylamide, (f) α-fluoroacrylamide, (g) α-cyanoacrylamide, (h) 4-(dimethylamino)-2-butenamide. Note that no transition states were obtained for the synclinal addition of methanethiolate to azetidinyl vinyl ketone and for both synclinal and antiperiplanar additions of methanethiolate to α-cyanoacrylamide. Hydrogen, carbon, nitrogen, oxygen, fluorine, and sulfur atoms are shown in white, gray, blue, red, green, and yellow, respectively. The partially formed bond for each transition state is shown as a black dotted line.

We now move onto the properties of the transition states, which are visualized in Figure 5. As can be seen by the Δ*G*^‡^ values in Table 1, the energetic barriers for synclinal addition are much lower than antiperiplanar addition. The observed trend shows that the reaction pathway for synclinal addition is much more energetically favorable than antiperiplanar additions, which has been reported in literature.^26,28^ We were unable to obtain the transition states for the synclinal addition of methanethiolate to azetidinyl vinyl ketone and for both synclinal and antiperiplanar additions of methanethiolate to cyanoacrylamide; in the former case, the transition state obtained from our calculations corresponded to pyramidalization of the nitrogen atom rather than the vibrational motion between the sulfur and β-carbon atoms. We were also unable to acquire correct transition states for the Θ_min_ ≈ 310° surfaces; given that Δ*G*_min_ for this surface is typically quite close to 0 kcal mol^–1^, it is possible that the reaction pathway is smoother without an energetic barrier to overcome, and there might not exist a distinct, well-defined transition state for this surface.

#### Effect of Solvation

All the calculations reported above were performed in the gas phase. The absence of solvation effects and protein-induced polarization means we were able to gain fundamental insights into the intrinsic conformational preferences of the reactants, which provides an important baseline for subsequent investigations of the influence of particular solvent environments. However, to study whether the trends observed herein persist in solution, we investigate the nucleophilic addition between methanethiolate and two of the warheads in Figure 2, namely acrylamide and azetidinyl vinyl ketone, within implicit protein and water environments. An implicit solvent model for water has been employed in previous studies of thio-Michael additions,^29^ though it can lead to different stabilizations of transition states and enolates than would be observed in a biological environment; for this reason, an implicit protein environment was also considered. The implicit solvent environment captures the impact due to the bulk properties of the medium.

**Figure 6:**
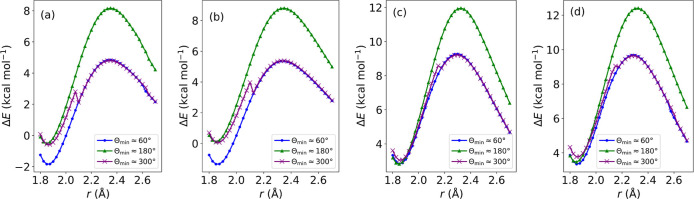
PES scans showing the relative energy, Δ*E*, with respect to the energies of the reactants for the S–C^β^ bond length, *r*, during the nucleophilic addition of methanethiolate to (a) acrylamide and (b) azetidinyl vinyl ketone within an implicit protein environment and to (c) acrylamide and (d) azetidinyl vinyl ketone within an implicit water environment. All values calculated at the ωB97X-D3(BJ)/aug-cc-pVTZ level.

PES scans, with *r* as the reaction coordinate, were performed to acquire reaction profiles in implicit protein and water (see Figure 6). Much like the gas-phase PES scans in Figure 4, there is an energetic minimum around *r* ≈ 1.85 Å for all the PES scans across all warheads. A point of discontinuity at *r* ≈ 2.0 Å for each of the PES scans corresponding to the Θ_min_ ≈ 300° surfaces can also be seen, with all the structures along the PES scan corresponding to the Θ_min_ ≈ 300° surface following that of the Θ_min_ ≈ 60° surface. One notable difference between the gas-phase and solvated calculations is that the enolates and transition states are higher in energy with respect to the energies of the reactants than in the gas phase.

Table 2 summarizes the structural and energetic characteristics of the enolate and their corresponding transition states in implicit protein and water. As can be seen, the energy barriers, Δ*G*^‡^, for antiperiplanar addition are over 2–3 kcal mol^–1^ higher than synclinal addition in both solvent environments. The energy barriers in solution, themselves, are significantly larger than in the gas phase; this is to be expected for a thio-Michael addition between a small ionic nucleophile and a neutral warhead.^24,29^ We also note that we were successful in obtaining solvated transition states for the synclinal addition of methanethiolate to azetidinyl vinyl ketone, unlike in the gas phase. This is in line with observations in the literature whereby transition states are easier to obtain within a solvent environment.^24,29^ The Δ*G*_min_ values for implicit protein in Table 2 corresponding to the Θ ≈ 60° surface are about 1.2 kcal mol^–1^ lower than antiperiplanar addition; however, the Δ*G*_min_ values for the Θ ≈ 180° and Θ ≈ 300° surfaces are very similar to each other. This is in contrast to the gas phase, where synclinal addition was more energetically favorable than antiperiplanar, as shown in Table 1. This can be rationalized by comparing the Δ*G*_solv_ values in Table 2. As can be seen, Δ*G*_solv_ for antiperiplanar addition is consistently the most negative among all three surfaces. This indicates that antiperiplanar addition is stabilized more by the solvent environment than synclinal addition in implicit protein. In implicit water, the Δ*G*_min_ values in Table 2, across all three surfaces, are very similar to each other. This is in contrast to the gas phase and implicit protein, where synclinal addition was more energetically favorable than antiperiplanar, as shown in Table 1. This can also be rationalized by comparing the Δ*G*_solv_ values in Table 2. As can be seen, Δ*G*_solv_ for antiperiplanar addition is consistently over 3 kcal mol^–1^ more negative than addition at Θ_min_ ≈ 60° in implicit water. This indicates that antiperiplanar addition is stabilized more by the water environment than synclinal addition.

**Table 2:** Structural and Energetic Characteristics of the Enolates and Transition States after the Nucleophilic Addition of Methanethiolate to Various Warheads in Three Different Conformations in Implicit Protein and Water Environments^a^

warhead	Θ_min_ (deg)	*r*_min_ (Å)	Δ*E*_min_	Δ*G*_min_	Δ*G*_solv_	Θ^‡^ (deg)	*r*^‡^ (Å)	(cm^–1^)	Δ*E*^‡^	Δ*G*^‡^
protein										
acrylamide	57.4	1.86	–1.85	+10.54	–44.28	15.5	2.35	294.7	+4.84	+16.75
	180.1	1.85	–0.51	+11.72	–46.58	186.7	2.35	321.8	+8.16	+19.20
	300.2	1.87	–0.60	+11.91	–45.30					
azetidinyl vinyl ketone	56.0	1.86	–1.36	+11.59	–42.43	15.3	2.34	284.7	+5.37	+17.20
	180.9	1.85	+0.11	+12.77	–44.32	169.8	2.35	314.9	+8.81	+20.20
	299.2	1.86	+0.05	+12.76	–43.69					
water										
acrylamide	59.6	1.86	+2.82	+15.06	–59.41	0.1	2.29	343.5	+9.27	+20.95
	180.9	1.85	+2.81	+14.97	–62.99	186.9	2.32	382.4	+11.96	+22.71
	297.6	1.86	+3.03	+15.50	–61.36					
azetidinyl vinyl ketone	57.4	1.86	+3.34	+16.34	–57.12	11.2	2.29	341.1	+9.68	+21.64
	180.8	1.85	+3.46	+16.28	–60.25	182.4	2.32	373.6	+12.41	+23.79
	297.1	1.86	+3.76	+16.36	–59.53					

a The following are included for the enolates: the C^α^–C^β^–S–C dihedral angles (Θ_min_), S–C^β^ bond lengths (*r*_min_), relative energies (Δ*E*_min_), relative Gibbs free energies (Δ*G*_min_), and Gibbs free energies of solvation (Δ*G*_solv_). The following are included for the transition states: the C^α^–C^β^–S–C dihedral angle (Θ^‡^), S–C^β^ bond lengths (*r*^‡^), magnitude of the frequencies of the sole imaginary mode , relative energies (Δ*E*^‡^), and relative Gibbs free energies (Δ*G*^‡^). Note that no transition states were obtained for the Θ_min_ ≈ 300° minima; see main text for details. All values were calculated at the ωB97X-D3(BJ)/aug-cc-pVTZ level and all energies (Δ*E*_min_, Δ*G*_min_, Δ*G*_solv_, Δ*E*^‡^, and Δ*G*^‡^) are in kilocalories per mole (kcal mol^–1^).

Despite the differences between gas-phase and implicit solvent environments, the greater energetic favorability of synclinal surfaces over antiperiplanar remains even with the inclusion of an implicit solvent.

### Torsional Transition States

**Figure 7:**
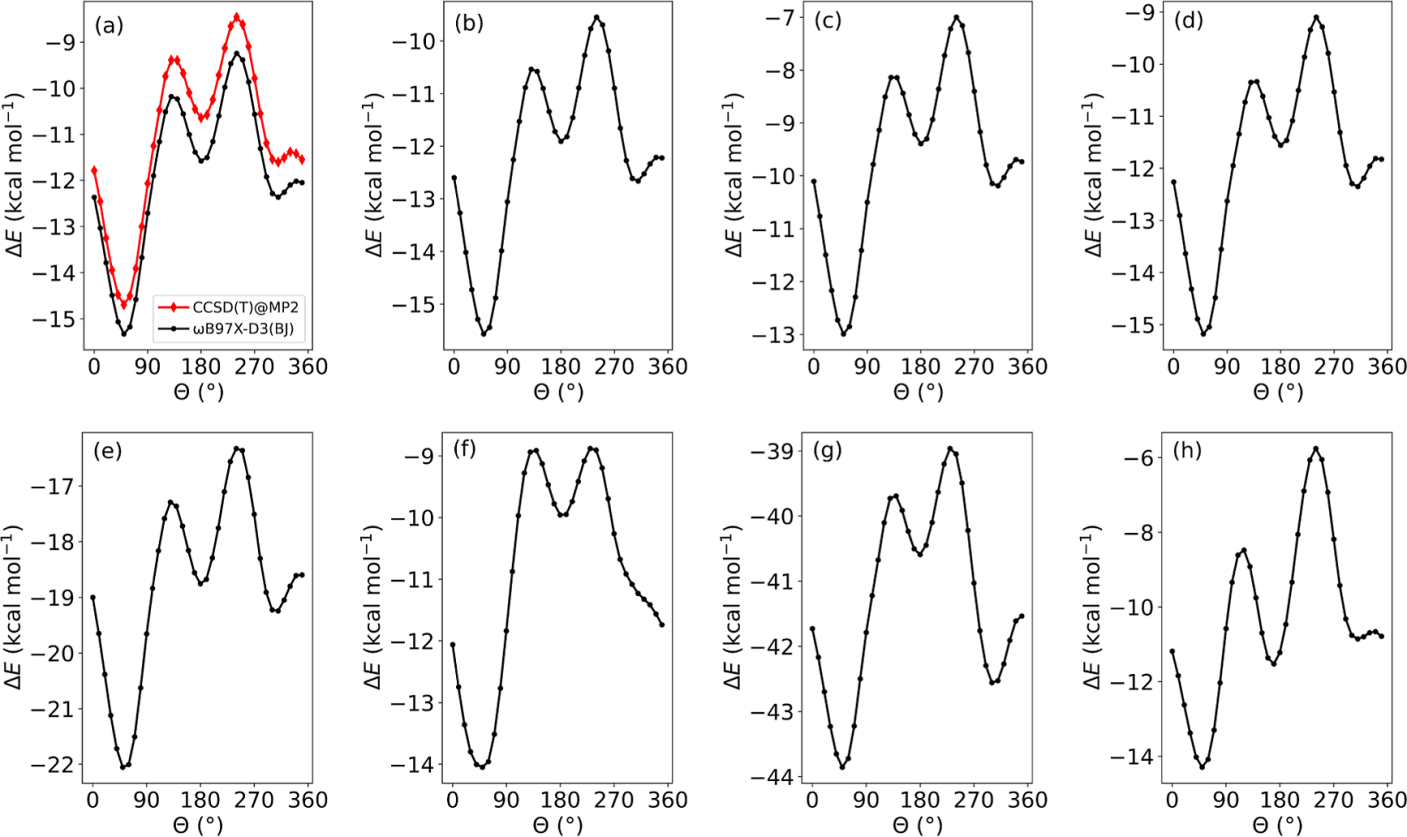
PES scans showing the relative energy, Δ*E*, with respect to the energies of the reactants for the C^α^–C^β^–S–C dihedral angle, Θ, during the nucleophilic addition of methanethiolate to (a) acrylamide, (b) azetidinyl vinyl ketone, (c) pyrrolidinyl vinyl ketone, (d) piperidinyl vinyl ketone, (e) *N*-phenylacrylamide, (f) α-fluoroacrylamide, (g) α-cyanoacrylamide, and (h) 4-(dimethylamino)-2-butenamide.

Having studied the reaction profiles of various thio-Michael additions, we now look into the energetic barriers associated between the identified enolate rotamers. PES scans, with Θ as the reaction coordinate, were conducted to identify torsional transition states (see Figure 7). The trends in Figure 7 are similar for each warhead; there are three energetic minima at Θ ≈ 50, 310, and 180° (from most to least stable, as determined by the magnitudes of Δ*E* values). These rotamers correspond to the three conformations shown in Figure 3 and verify the results shown above. As can be seen in Figure 7a, the Θ-dependent PES curves calculated with ωB97X-D3(BJ) remain accurate with respect to CCSD(T)@MP2, with a mean absolute error of 1.15 kcal mol^–1^ between the two methods. The CCSD(T) calculations were found to have a mean average *T*_1_ diagnostic value of 0.0146 and a standard deviation of 1.34 × 10^–4^, indicating their reliability. The dependence of *T*_1_ on Θ can be seen in Figures S4(c). As can be seen in Figure 7f, there is no minimum at Θ ≈ 310° for α-fluoroacrylamide, while the Δ*E* values for α-cyanoacrylamide are much more negative than the other warheads, as can be seen in Figure 7g.

Table 3 summarizes the structural and energetic characteristics of torsional transition states. Across the warheads, torsional transition states typically occur at dihedral angles of Θ^‡^ ≈ 134° (between Θ_min_ ≈ 50° and Θ_min_ ≈ 180°), 242° (between Θ_min_ ≈ 180° and Θ_min_ ≈ 310°), and 345° (between Θ_min_ ≈ 310° and Θ_min_ ≈ 50°). The torsional energetic barriers required to rotate from Θ_min_ ≈ 50° to Θ_min_ ≈ 180° or Θ_min_ ≈ 310° are Δ*G*_Θ↑_^‡^≈ 5.0 kcal mol^–1^ and Δ*G*_Θ↓_^‡^≈ 3.0 kcal mol^–1^, respectively, further corroborating the greater stability of the Θ_min_ ≈ 50° rotamer. In comparison, the torsional energetic barriers required to rotate from Θ_min_ ≈ 130° to Θ_min_ ≈ 310° or Θ_min_ ≈ 50° are Δ*G*_Θ↑_^‡^≈ 3.0 kcal mol^–1^ and Δ*G*_Θ↓_^‡^≈ 2.0 kcal mol^–1^, respectively. For Θ_min_ ≈ 310°, the torsional energetic barriers required to rotate to Θ_min_ ≈ 50° and Θ_min_ ≈ 130° are Δ*G*_Θ↑_^‡^≈ 1.0 kcal mol^–1^ and Δ*G*_Θ↓_^‡^≈ 2.7 kcal mol^–1^, respectively. Noticeably, the energetic barrier to rotate from Θ_min_ ≈ 130° to Θ_min_ ≈ 50° is quite low, while the inverse torsion is 2.0 kcal mol^–1^ higher, indicating the greater stability of the Θ_min_ ≈ 50° rotamer.

**Table 3:** Structural and Energetic Characteristics of Torsional Transition States^a^

warhead	Θ^‡^ (deg)	(cm^–1^)	Δ*G*_Θ↑_^‡^ (kcal mol^–1^)	Δ*G*_Θ↓_^‡^ (kcal mol^–1^)
acrylamide	133.5	72.6	5.32	3.50
	241.7	76.8	2.62	1.83
	344.0	83.7	1.11	2.93
azetidinyl vinyl ketone	134.0	65.2	4.89	3.30
	241.1	79.5	2.84	1.83
	345.0	59.4	0.88	2.47
pyrrolidinyl vinyl ketone	133.3	64.2	5.18	2.54
	241.6	72.9	3.09	2.33
	345.3	80.8	0.10	2.74
piperidinyl vinyl ketone	134.4	65.7	4.96	3.06
	242.6	65.0	2.91	1.98
	344.6	45.7	0.76	2.66
*N*-phenylacrylamide	133.2	58.5	+4.82	3.42
	240.7	72.7	3.09	2.12
	345.0	63.5	1.37	2.76
α-fluoroacrylamide	136.2	65.3	5.25	5.12
	234.6	53.7	1.79	1.92
α-cyanoacrylamide	136.2	60.8	4.53	2.95
	230.8	68.3	2.24	1.45
	349.0	65.6	1.48	3.06
4-(dimethylamino)-2-butenamide	117.1	81.8	5.74	3.34
	240.3	97.6	5.98	2.98
	337.6	51.3	0.66	3.06

a Included are the: C^α^–C^β^–S–C dihedral angles (Θ^‡^), magnitude of the frequencies of the sole imaginary mode , and torsional energetic barriers, and Δ*G*_Θ↑_^‡^, of the transition states during the nucleophilic addition of methanethiolate to various warheads. All values calculated at the ωB97X-D3(BJ)/aug-cc-pVTZ level.

### Non-covalent Interactions

To understand the observed stability trend between the enolate rotamers, the electronic structures of the individual rotamers were further investigated. The oppositely charged nature of intermolecular species could give rise to some favorable noncovalent electrostatic interactions, if permitted conformationally. First, the Mulliken charge^59^ distributions within each rotamer were extracted (see Figures S6–S13 in the Supporting Information). As the aug-cc-pVTZ basis set is fairly complete, there is some ambiguity within our Mulliken analyses as it is not *a priori* clear which electrons should count toward the basis functions of one atom over another. However, we only use Mulliken analysis as a qualitative indicator of trends across the various systems. For each rotamer, we observe that every hydrogen atom has a positive Mulliken charge, whereas the oxygen atom and every carbon atom have negative Mulliken charges, with the exceptions of the former-carbonyl carbon atom in every warhead and the α-carbon atom in α-fluoroacrylamide. The latter is to be expected due to the much higher electronegativity of fluorine as compared to carbon,^60^ which thus renders the α-carbon slightly positive (Mulliken charges of +0.47 *e* and +0.53 *e* for the Θ_min_ ≈ 50 and 180° rotamers, respectively, as shown in Figure S11). Figure S12 also explains the observation of the Δ*E* values being much more negative for α-cyanoacrylamide than other warheads. The highly negative Mulliken charge on the cyano-nitrogen indicates significant electron-withdrawing effects which stabilize the enolate intermediate, resulting in lower energies due to increased stabilization of the electron density around the cyano group.

**Figure 8:**
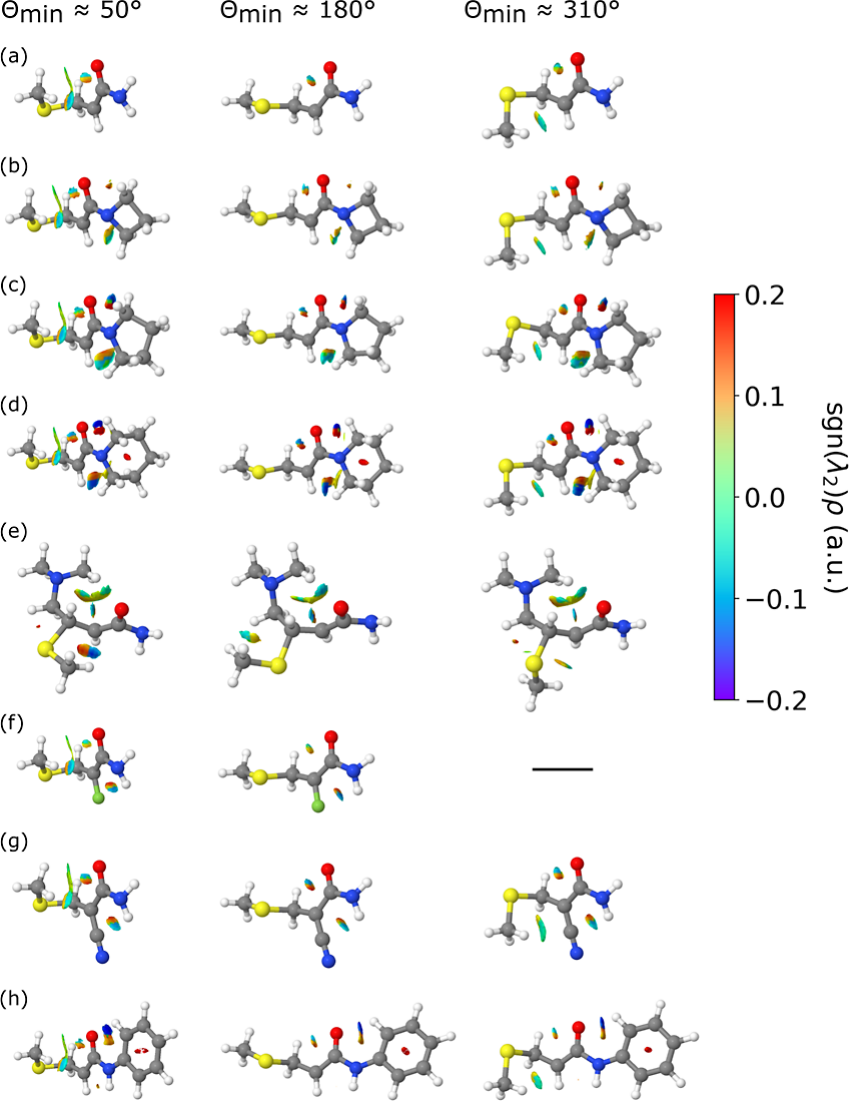
Gradient isosurfaces of various enolate rotamers, with Θ_min_ ≈ 50° (first column), 180° (second column) and 310° (third column), after the nucleophilic addition of methanethiolate to (a) acrylamide, (b) azetidinyl vinyl ketone, (c) pyrrolidinyl vinyl ketone, (d) piperidinyl vinyl ketone, (e) *N*-phenylacrylamide, (f) α-fluoroacrylamide, (g) α-cyanoacrylamide, and (h) 4-(dimethylamino)-2-butenamide. A colorbar is shown with values of sgn(λ_2_)ρ, which is the sign of the second Hessian eigenvalue (λ_2_) multiplied by the electron density (ρ). Blue surfaces indicate strong attraction, green surfaces indicate weak interactions, and red surfaces indicate repulsion and strong nonbonded overlap. Hydrogen, carbon, nitrogen, oxygen, fluorine, and sulfur atoms are shown in white, gray, blue, red, green, and yellow, respectively. Note that no Θ_min_ ≈ 310° minimum was observed for the α-fluoroacrylamide warhead.

To investigate the relative stability further among the enolate rotamers, interaction regions^54^ were calculated using the NCIPLOT^53^ program and can be seen in Figures S14 and S15. The noncovalent regions in Figures S14 and S15 can be identified by the low-density, low-gradient spikes, which are mostly bound by ρ < 0.03 au; this bound was therefore chosen for the visualization of noncovalent interactions. Figure 8 visualizes all the rotamers and their gradient isosurfaces, which allow for a rich visualization of the noncovalent interactions as broad regions of real space,^54^ using the ρ < 0.03 au bound for the density. We first focus on analyzing the noncovalent interactions for all warheads apart from α-fluoroacrylamide.

As can be seen by the interactions between the original nucleophile and the main body of the warhead (i.e., not the R group in Figures 1 and 3), the isosurfaces for the Θ_min_ ≈ 50° rotamer have three attractive interactions: between a slightly positively charged C^β^-bonded hydrogen and the negatively charged oxygen, between a slightly positively charged methyl-hydrogen and the slightly negatively charged α-carbon, and a weak attraction between another methyl-hydrogen and the oxygen. Some repulsion is observed also between the aforementioned C^β^-bonded hydrogen and the former-carbonyl carbon, and between the methyl- and α-carbons, which is to be expected due to their like charges. In contrast, the Θ_min_ ≈ 180° rotamer only has the attractive and repulsive interactions between the C^β^-bonded hydrogen and the former-carbonyl group; the CH_3_S group is oriented away from the main body of the warhead, so the other noncovalent interactions between the CH_3_S group and the main warhead are not present in this rotamer. Finally, the Θ_min_ ≈ 310° rotamer possesses the same noncovalent interactions as the other synclinal rotamer apart from the weak attraction between the methyl-hydrogen and the oxygen, which is due to the conformation of the methyl group with respect to the main warhead. From Figure 8, it can be seen that synclinal additions are more stable over antiperiplanar due to the magnitude of attractive noncovalent interactions between the CH_3_S group and the warhead. This is in contrast to previous rationalizations, which determine the greater stability of synclinal rotamers to be due to the lone pairs on the sulfur being located further away from the C^α^=C^β^ electron density and avoiding repulsive interactions with the lone pairs on the oxygen.^29^

The noncovalent interactions can also rationalize why there is no Θ_min_ ≈ 310° minimum for the α-fluoroacrylamide warhead. As mentioned above, the presence of a highly electronegative species like fluorine bonded to the α-carbon renders the α-carbon slightly positive. However, as the methyl-hydrogen atoms are also slightly positively charged, as shown in Figure S11, there is no attractive force between the methyl-hydrogen and the α-carbon that is required for the formation of the Θ_min_ ≈ 310° conformation.

We also observe that the magnitude of noncovalent interactions in the terminal region of the enolate unsurprisingly increases as the warhead size increases. This can be seen in Figures S14 and 8, where the low-density, low-gradient spikes within the noncovalent interaction regions become denser and the magnitude of the gradient isosurfaces increases, respectively, as the warhead size increases. As the ring size of the terminal group increases, the two nitrogen-bonded methylene (CH_2_) groups are pulled closer toward the oxygen and the α-carbon in the main warhead body. This results in repulsion between the oxygen and a carbon in the terminal group and attraction between the oxygen and a hydrogen in the terminal group, and these interactions strengthen as the ring size of the terminal group increases. Furthermore, the gradient isosurfaces show that increasing the ring size of the nitrogen heterocycle has no significant effect on the noncovalent interactions experienced between the CH_3_S group and the warhead. Additional noncovalent interactions can also be seen in Figure 8e between one of the methyl (CH_3_) groups and the main body of the 4-(dimethylamino)-2-butenamide warhead. An area of nonbonded overlap located at the center of the piperidinyl and phenyl rings can also be seen in Figure 8d and h. The stability trend observed between the three rotamers can be rationalized by the magnitude of attractive noncovalent interactions that are permitted by the orientation of the nucleophile with respect to the main warhead body.

## Conclusion

Using quantum chemical methods, we have characterized the rotameric behavior in the Michael addition of methanethiolate, an archetypal nucleophile, to eight warheads that are representative of small-molecule ligands that have been promising for the covalent inhibition of CDK12 for DM1 treatment. Three rotamers were generally identified to be at energetic minima, one of them being an antiperiplanar addition and the other two being synclinal. The second synclinal minimum has not been reported in previous studies of thio-Michael additions. Investigation of the noncovalent interactions within these rotamers revealed that synclinal addition at a C^α^–C^β^–S–C dihedral angle of around 50° has the highest magnitude of attractive interactions, enabled by the relative orientations of the reacting fragments, which stabilize the rotamer. Other rotamers have fewer favorable noncovalent interactions due to their conformations, which is why they are less energetically favorable. By characterizing the transition states associated with the nucleophilic addition process, we also observe that synclinal addition has lower energetic barriers and a preferred reaction pathway than anticlinal addition. The trends observed in the gas phase are mostly conserved in solvent too; while the implicit protein environment was found to stabilize antiperiplanar addition more than synclinal, the energy barriers associated with antiperiplanar addition in protein were still calculated to be greater than for synclinal.

## Data Availability

The quantum chemical calculations were performed using the ORCA software package, which is freely available to academic users. Input and output files for all calculations have been uploaded as a data set to the NOMAD electronic structure data repository and are freely available under 10.17172/NOMAD/2024.04.18-1.^61^

## Supporting Information Available

Supporting Information: *T*_1_ diagnostics of CCSD(T) calculations, enolate stereoisomers and conformations, Mulliken charge distributions of enolate rotamers, noncovalent interaction plots, and *r*-dependent PES scans (PDF)
