# Supplementary material for: Quantum Chemical Characterization of Rotamerism in Thio-Michael Additions for Targeted Covalent Inhibitors
Source: J Chem Inf Model. 2024 Sep 12;64(19):7687–97. doi: 10.1021/acs.jcim.4c01379 (PMC11480980; doi:10.1021/acs.jcim.4c01379)
Supplement: Supplementary file 1 — ci4c01379_si_001.pdf [file ci4c01379_si_001.pdf]

Supporting Information: 'Quantum Chemical Characterization of Rotamerism in Thio-Michael Additions for Targeted Covalent Inhibitors'

Shayantan Chaudhuri, David M. Rogers, Christopher J. Hayes,* Katherine Inzani,* and Jonathan D. Hirst*

School of Chemistry, University of Nottingham, Nottingham, NG7 2RD, United Kingdom

E-mail: chris.hayes@nottingham.ac.uk; katherine.inzani1@nottingham.ac.uk; jonathan.hirst@nottingham.ac.uk

1 Potential Energy Surface Scans for S–C$^{\beta}$ Bond Length

As shown in Figures S1(a) and S1(b), the r-dependent PES curves calculated with ωB97X-D3(BJ) remain accurate with respect to CCSD(T)@MP2, with a mean absolute error of 1.15 kcal mol^{-1} between the two methods. Both sets of CCSD(T) calculations had a mean average T_1 diagnostic value[S1] of 0.016 and a standard deviation of 0.001, indicating their reliability. The dependence of T_1 on r can be seen in Figures S4(a) and S4(b).

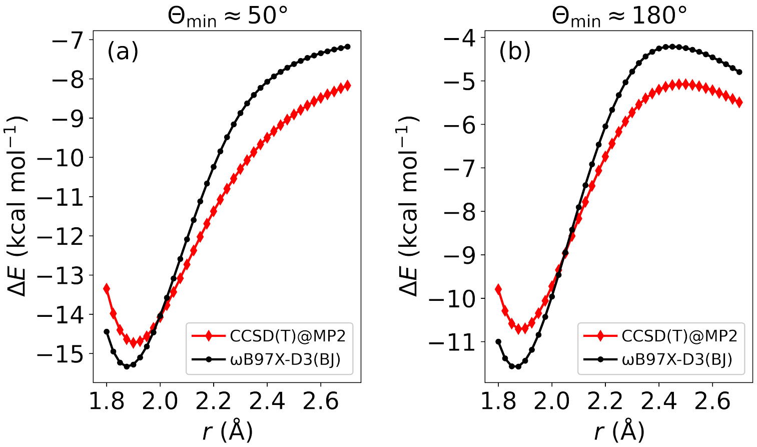

Figure S1. Potential energy surface scans showing the relative energy, ΔE, with respect to the energies of the reactants for the (a) synclinal and (b) antiperiplanar nucleophilic addition of methanethiolate to acrylamide, as a function of the S–C$^{\beta}$ bond length, r.

Figures S2 and S3 show the change in the Mulliken charge[S2] of the sulfur, q_S during the PES scans. As can be seen, in all cases, $q_S \to -1\,e$ for $r > r_{min}$, confirming the S–C$^{\beta}$ bond fission process to be heterolytic and that the negative charge is localized on the sulfur atom as r increases, as expected. There is a point of discontinuity at $r = 1.825$ Å; this is likely due to overlap between the atomic orbitals of the sulfur and β-carbon atoms at such low r, and the partial transfer of electron density from the β-carbon atom to the sulfur atom result in an increase in the electron density around the sulfur atom, causing q_S to become more negative.

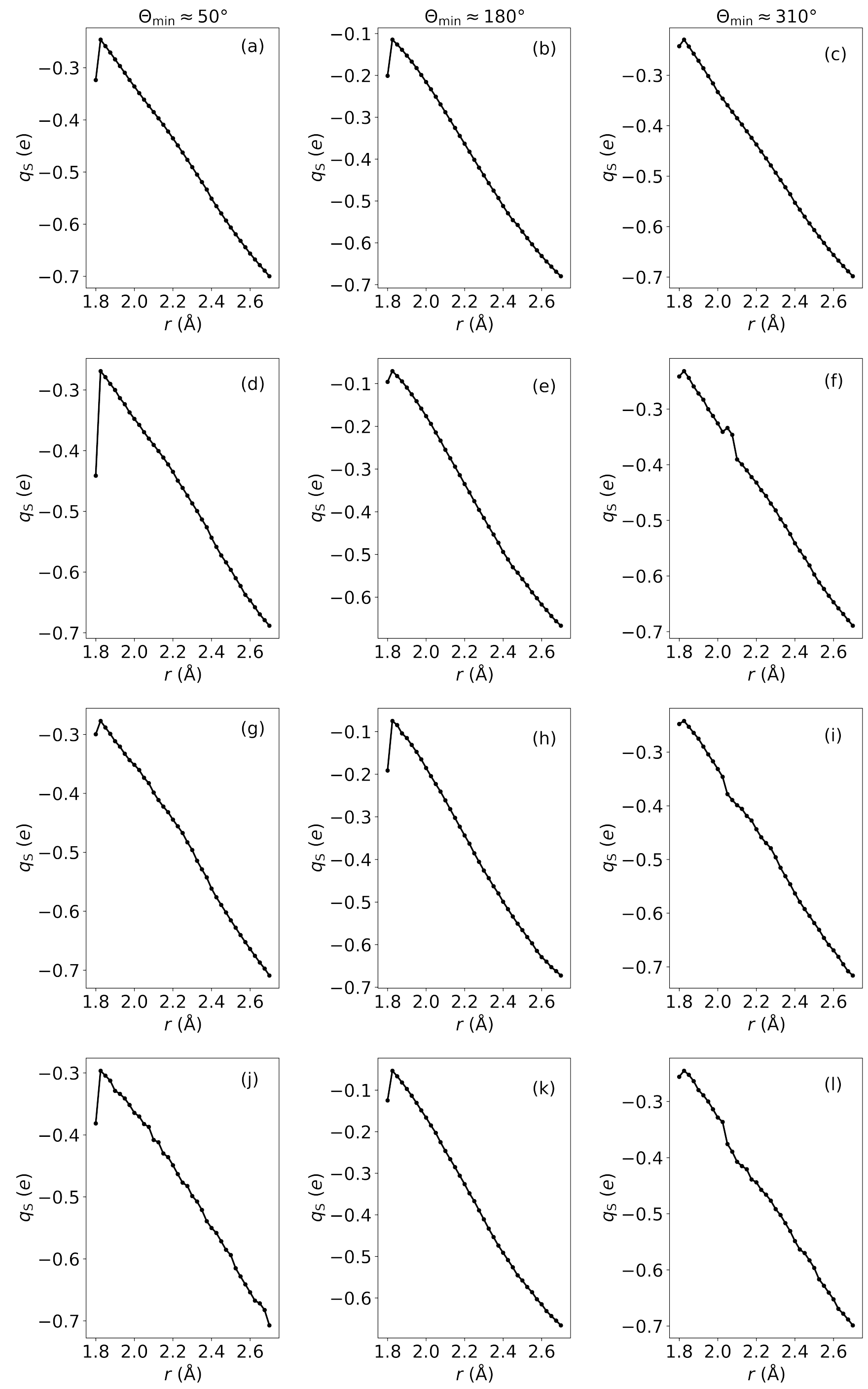

Figure S2. Mulliken charge of the sulfur, q_S, as a function of r for the addition of methanethiolate to (a)–(c) acrylamide, (d)–(f) azetidinyl vinyl ketone, (g)–(i) pyrrolidinyl vinyl ketone, and (j)–(l) piperidinyl vinyl ketone, with $\Theta_{min} \approx 50°$ (first column), 180° (second column) and 310° (third column).

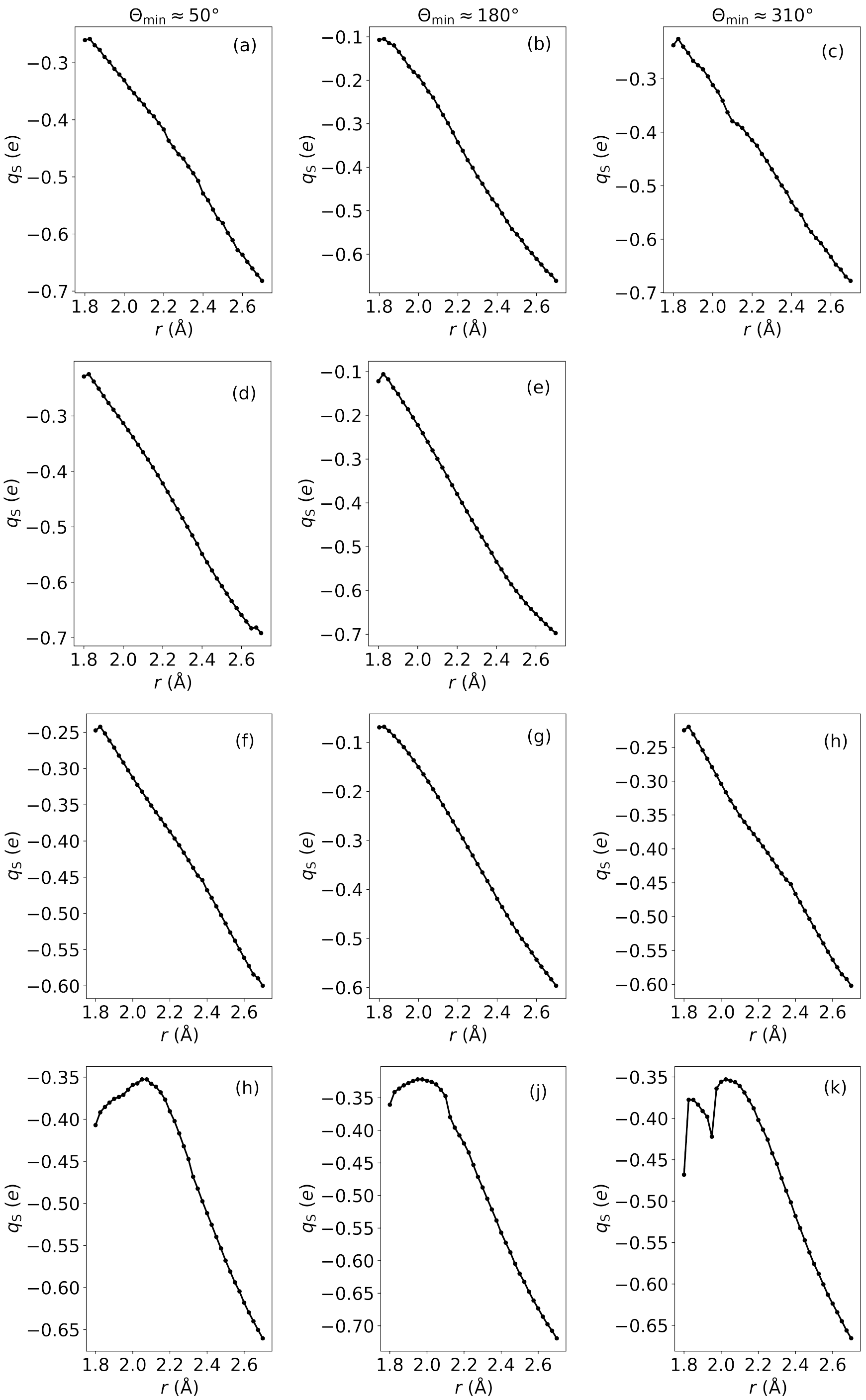

Figure S3. Mulliken charge of the sulfur, q_{S}, as a function of r for the addition of methanethiolate to (a)–(c) *N*-phenylacrylamide, (d)–(e) α-fluoroacrylamide, (f)–(h) α-cyanoacrylamide, and (i)–(k) 4-(dimethylamino)-2-butenamide, with $\Theta_{\mathrm{min}} \approx 50^\circ$ (first column), 180° (second column) and 310° (third column).

2 T_1 Diagnostics of CCSD(T) Calculations

As can be seen in Figure S4, the CCSD(T)[S3] calculations performed herein can be deemed to be reliable as $T_1 < 0.02$ in all cases.[S4]

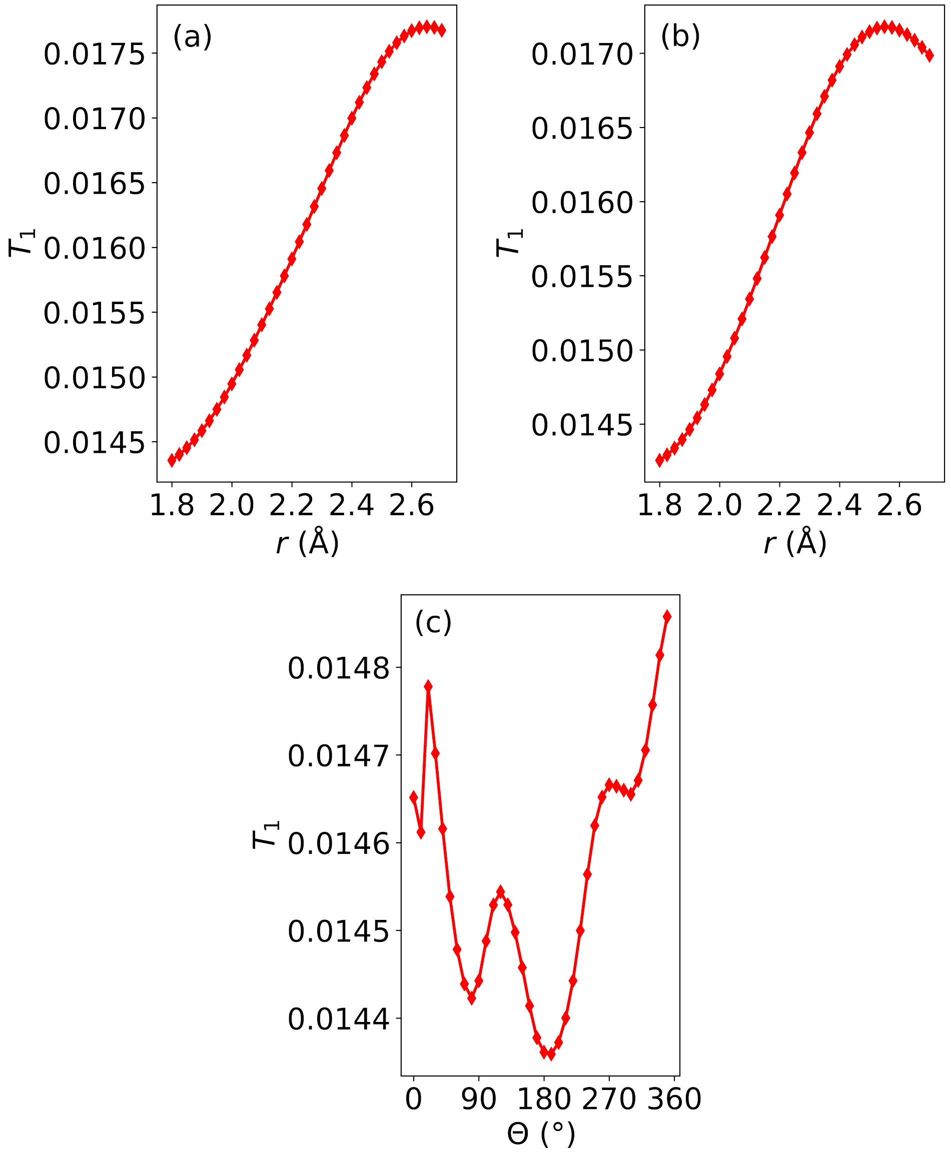

Figure S4. T_1 diagnostic values of the CCSD(T) calculations performed for the nucleophilic addition of methanethiolate to acrylamide. Shown are (b) T_1 values of the r-dependent PES scan with CCSD(T) shown in Figure S1(a), (c) T_1 values of the r-dependent PES scan with CCSD(T) shown in Figure S1(b), and (a) T_1 values of the Θ-dependent PES scan with CCSD(T) shown in Figure 6(a).

3 Enolate Stereoisomers and Conformations

To ensure the most stable enolate form was considered, the energies of other stereoisomers and conformations were compared. This was done for the synclinal addition of methanethiolate to acrylamide. The most stable form, visualized in Figure S5(a), was determined to be the *Z*-stereoisomer with pyramidalization at the nitrogen such that its lone pair is on the same side of the warhead as the CH_3S group. This can be seen in Table S1, where this form has the most negative absolute energy and Gibbs free energy.

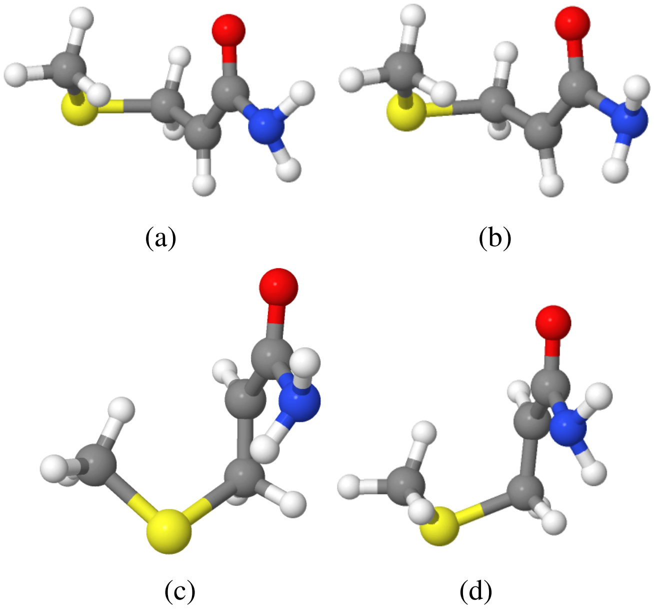

Figure S5. Visualizations of the various enolate forms after the synclinal nucleophilic addition of methanethiolate to acrylamide. Shown in (a) and (b) are the *Z*-stereoisomers with different pyramidalizations at the nitrogen, and shown in (c) and (d) are the *E*-stereoisomers with different pyramidalizations at the nitrogen. Hydrogen, carbon, nitrogen, oxygen, and sulfur atoms are shown in white, gray, blue, red, and yellow, respectively.

Table S1. Absolute energies and Gibbs free energies of various enolate forms after the synclinal addition of methanethiolate to acrylamide. The letters in the first column correspond with Figure S5. The most stable form is shown in bold.

Enolate	Energy (Ha)	Gibbs Free Energy (Ha)
**(a)**	**−685.6925**	**−685.6072**
(b)	−685.6922	−685.6071
(c)	−685.6910	−685.6056
(d)	−685.6885	−685.6033

4 Mulliken Charge Distributions of Enolate Rotamers

Figures S6–S13 show the Mulliken charge[S2] distributions for the various enolate rotamers.

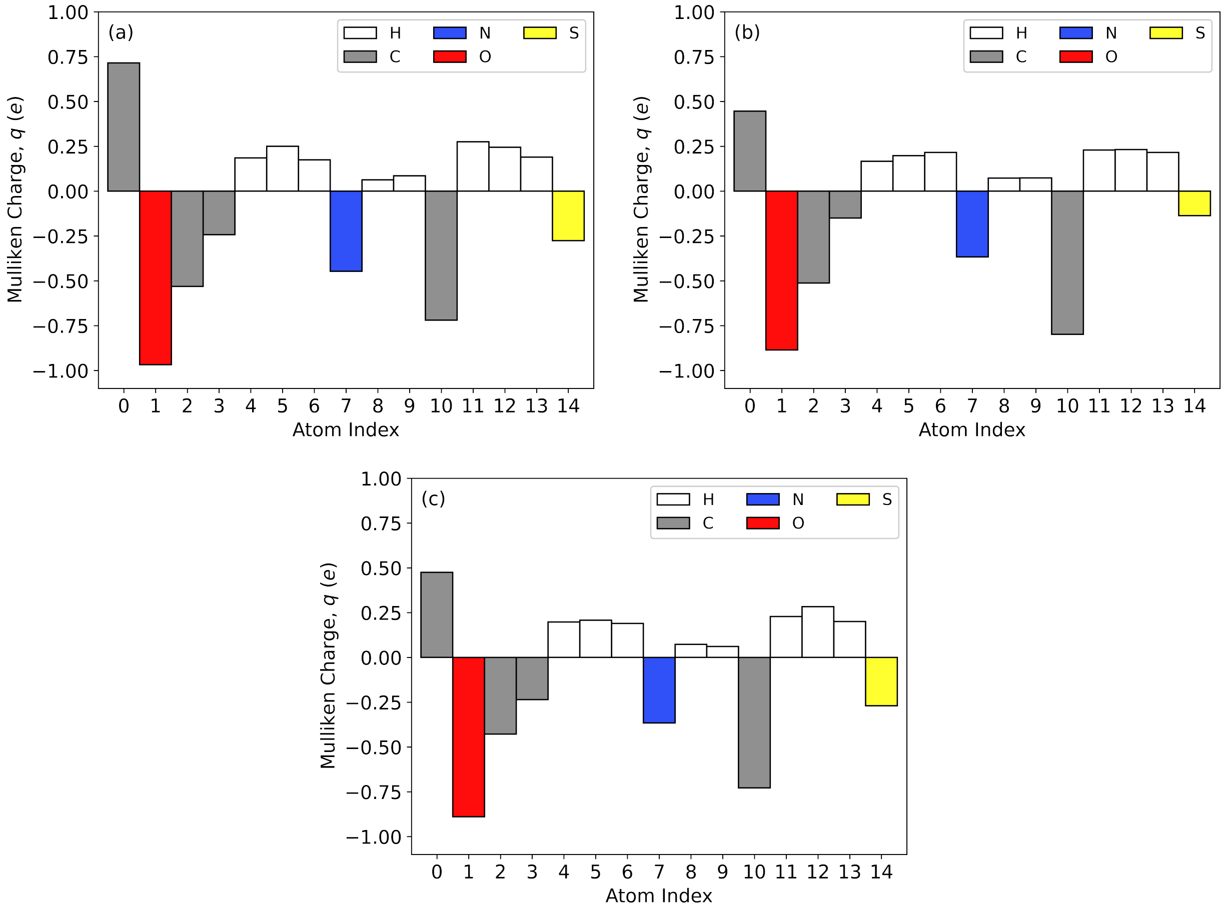

Figure S6. Mulliken charge distributions in the three enolate rotamers after the addition of methanethiolate to acrylamide. Shown are (a) $\Theta_{\text{min}} = 51.7°$, (b) $\Theta_{\text{min}} = 182.0°$, and (c) $\Theta_{\text{min}} = 309.0°$. Here, indices 0 and 1 are the carbonyl (C=O) group; indices 2 and 3 are the α- and β-carbons, respectively; index 4 is the hydrogen bonded to the α-carbon; indices 5–6 are the hydrogen atoms bonded to the β-carbon; indices 7–9 are the amino (NH_2) group; indices 10–13 are the methyl (CH_3) group; and index 14 is the sulfur.

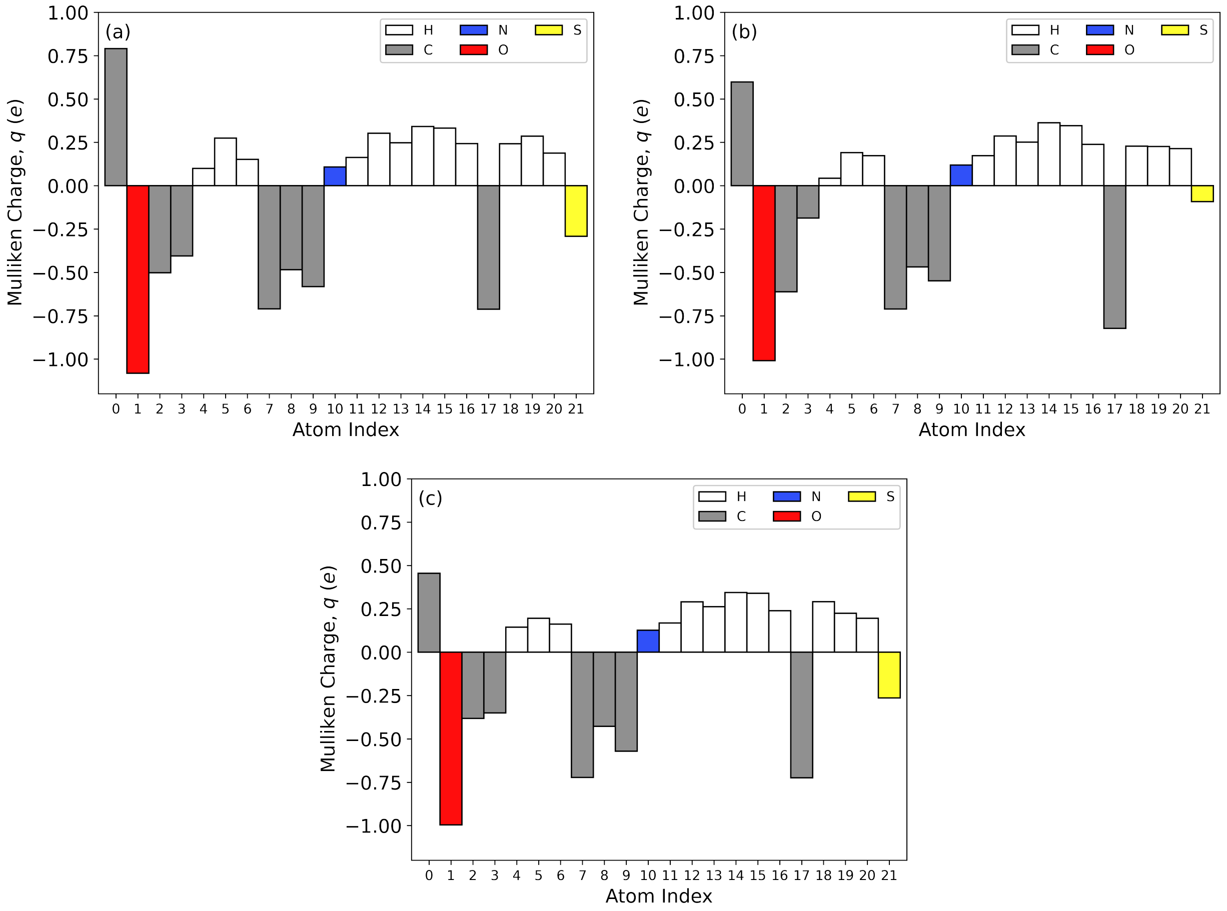

Figure S7. Mulliken charge distributions in the three enolate rotamers after the addition of methanethiolate to azetidinyl vinyl ketone. Shown are (a) $\Theta_{\mathrm{min}} = 51.7°$, (b) $\Theta_{\mathrm{min}} = 183.9°$, and (c) $\Theta_{\mathrm{min}} = 305.6°$. Here, indices 0 and 1 are the carbonyl (C=O) group; indices 2 and 3 are the α- and β-carbons, respectively; index 4 is the hydrogen bonded to the α-carbon; indices 5 and 6 are the hydrogen atoms bonded to the β-carbon; indices 7–16 are the azetidinyl $[N(CH_2)_3]$ group; indices 17–20 are the methyl (CH_3) group; and index 21 is the sulfur.

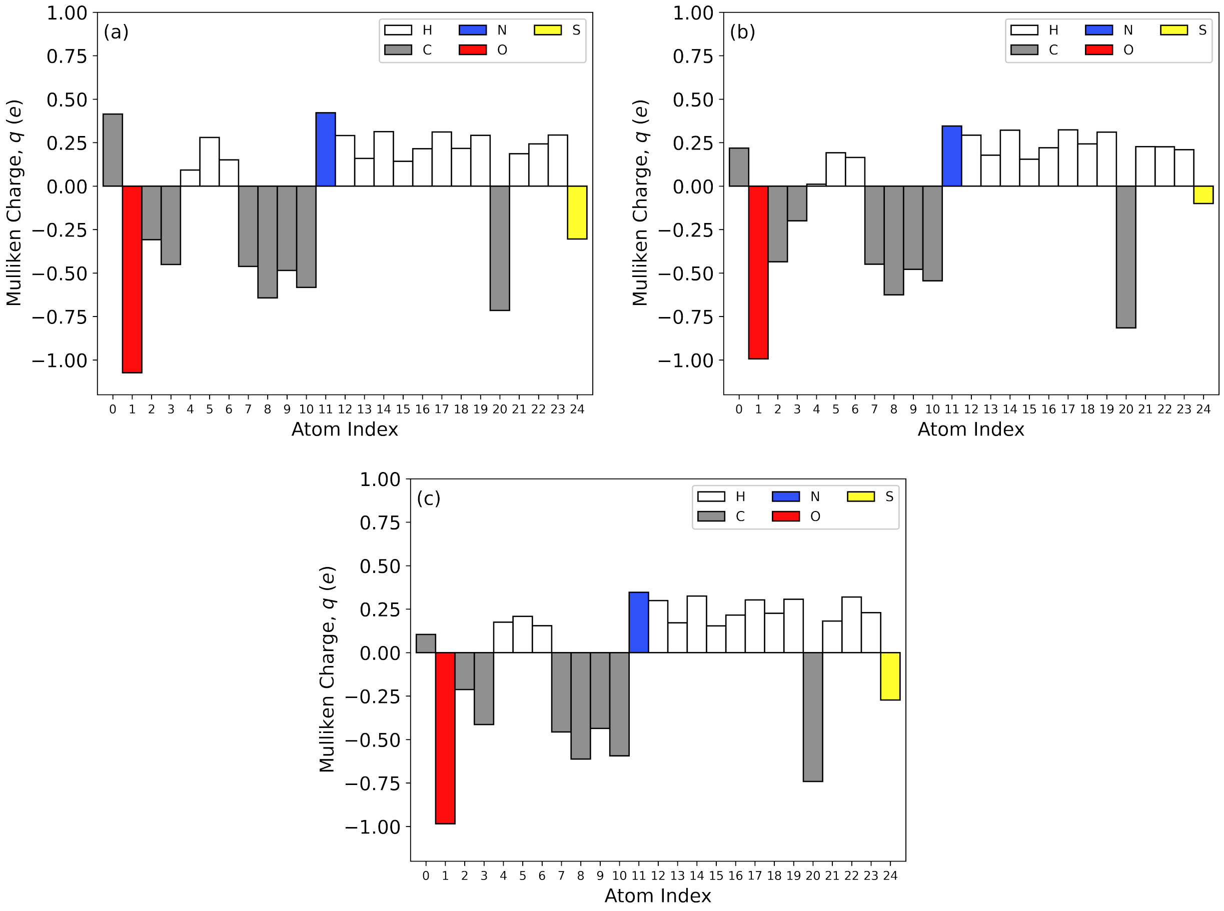

Figure S8. Mulliken charge distributions in the three enolate rotamers after the addition of methanethiolate to pyrrolidinyl vinyl ketone. Shown are (a) $\Theta_{\text{min}} = 51.3°$, (b) $\Theta_{\text{min}} = 183.2°$, and (c) $\Theta_{\text{min}} = 307.6°$. Here, indices 0 and 1 are the carbonyl (C=O) group; indices 2 and 3 are the α- and β-carbons, respectively; index 4 is the hydrogen bonded to the α-carbon; indices 5 and 6 are the hydrogen atoms bonded to the β-carbon; indices 7–19 are the pyrrolidinyl [$N(CH_2)_4$] group; indices 20–23 are the methyl (CH_3) group; and index 24 is the sulfur.

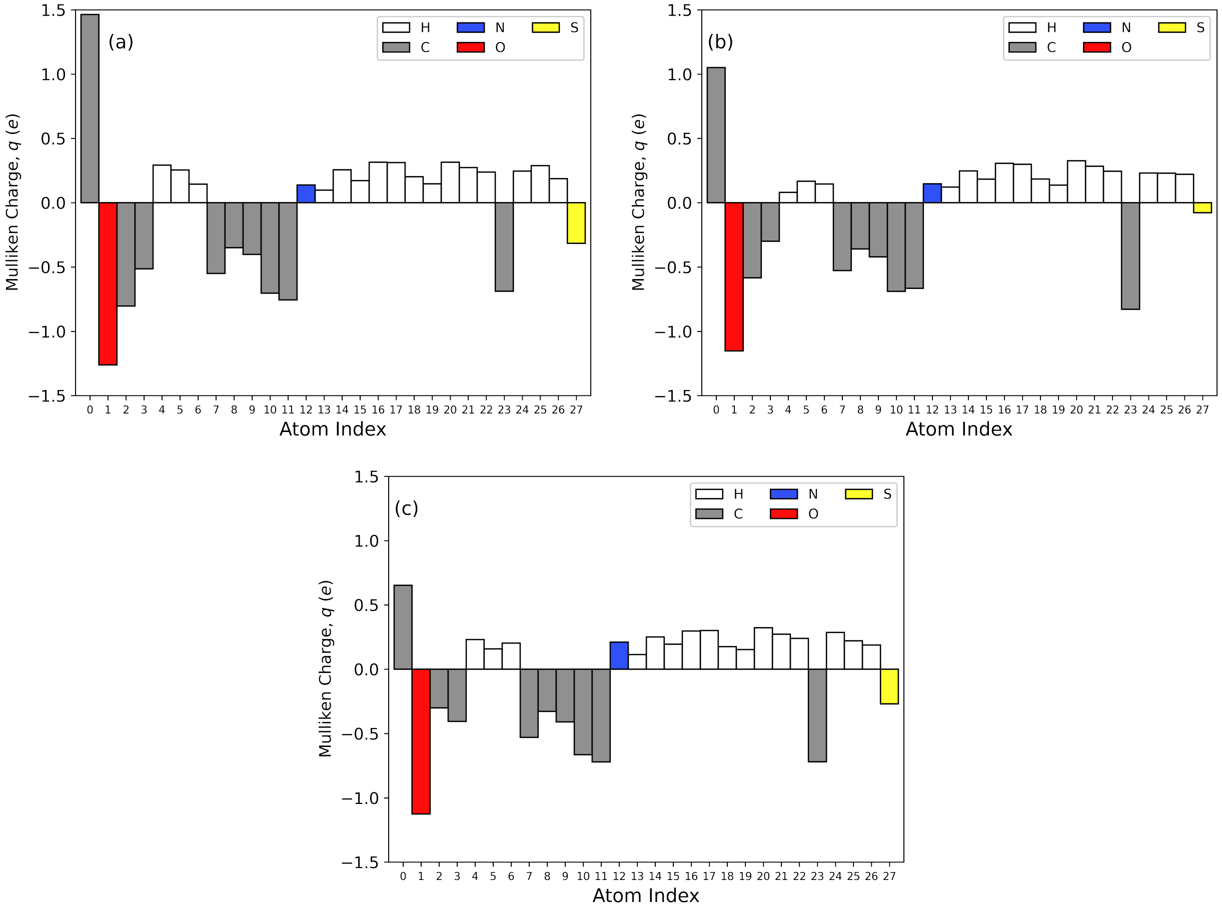

Figure S9. Mulliken charge distributions in the three enolate rotamers after the addition of methanethiolate to piperidinyl vinyl ketone. Shown are (a) $\Theta_{min} = 52.9°$, (b) $\Theta_{min} = 182.7°$, and (c) $\Theta_{min} = 306.9°$. Here, indices 0 and 1 are the carbonyl (C=O) group; indices 2 and 3 are the α- and β-carbons, respectively; index 4 is the hydrogen bonded to the α-carbon; indices 5 and 6 are the hydrogen atoms bonded to the β-carbon; indices 7–22 are the piperidinyl [$N(CH_2)_4$] group; and indices 23–26 are the methyl (CH_3) group; and index 27 is the sulfur.

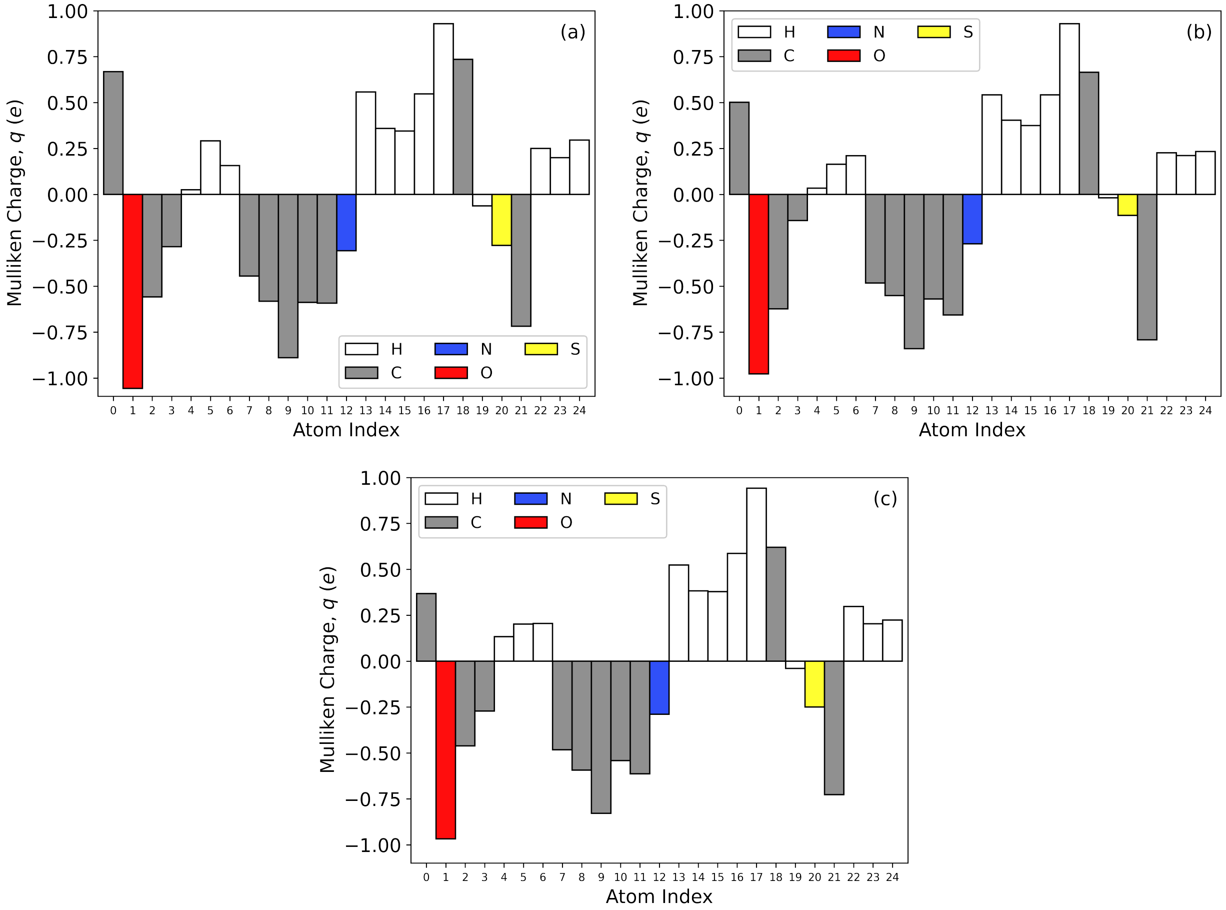

Figure S10. Mulliken charge distributions in the three enolate rotamers after the addition of methanethiolate to *N*-phenylacrylamide. Shown are (a) $\Theta_{\mathrm{min}} = 54.3^\circ$, (b) $\Theta_{\mathrm{min}} = 181.3^\circ$, and (c) $\Theta_{\mathrm{min}} = 304.7^\circ$. Here, indices 0 and 1 are the carbonyl (C=O) group; indices 2 and 3 are the α- and β-carbons, respectively; index 4 is the hydrogen bonded to the α-carbon; indices 5 and 6 are the hydrogen atoms bonded to the β-carbon; indices 7–11 and 13–17 are the phenyl (C_6H_5) group; indices 12 and 19 are the nitrogen and the hydrogen bonded to the nitrogen, respectively; index 20 is the sulfur; and indices 21–24 are the methyl (CH_3) group.

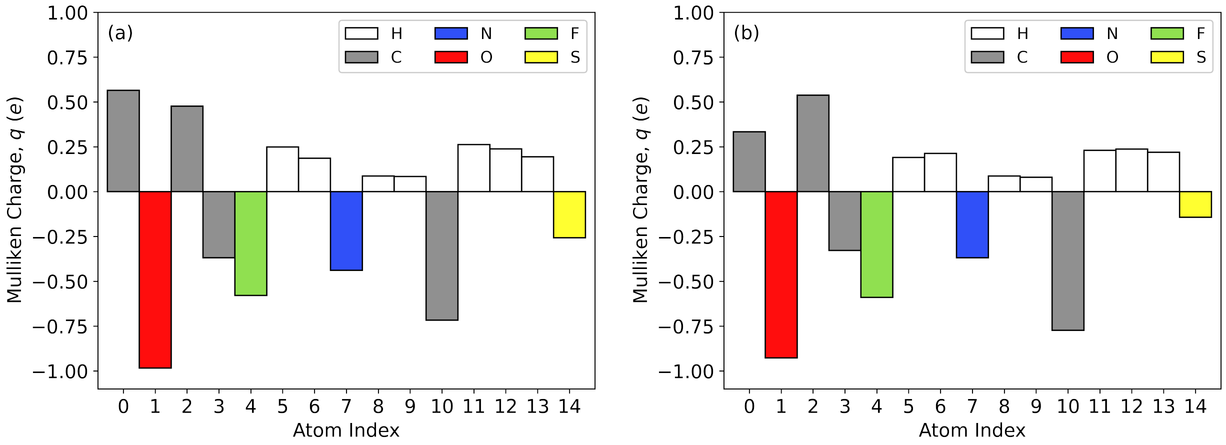

Figure S11. Mulliken charge distributions in the two enolate rotamers after the addition of methanethiolate to α-fluoroacrylamide. Shown are (a) $\Theta_{min} = 51.4°$, and (b) $\Theta_{min} = 183.2°$. Here, indices 0 and 1 are the carbonyl (C=O) group; indices 2 and 3 are the α- and β-carbons, respectively; index 4 is the fluorine bonded to the α-carbon; indices 5–6 are the hydrogen atoms bonded to the β-carbon; indices 7–9 are the amino (NH_2) group; indices 10–13 are the methyl (CH_3) group; and index 14 is the sulfur.

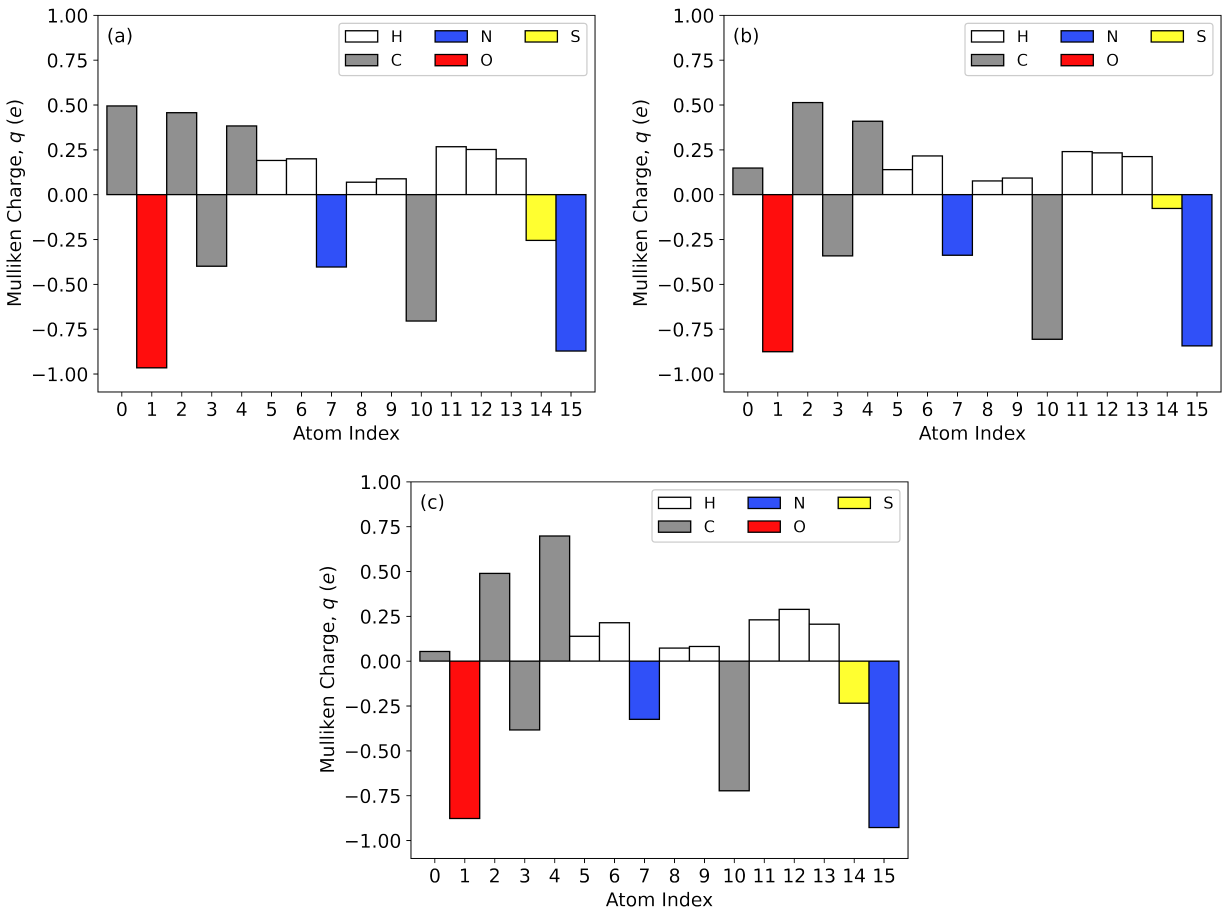

Figure S12. Mulliken charge distributions in the three enolate rotamers after the addition of methanethiolate to α-cyanoacrylamide. Shown are (a) $\Theta_{\mathrm{min}} = 51.0°$, (b) $\Theta_{\mathrm{min}} = 177.9°$, and (c) $\Theta_{\mathrm{min}} = 303.2°$. Here, indices 0 and 1 are the carbonyl (C=O) group; indices 2 and 3 are the α- and β-carbons, respectively; indices 4 and 15 are the cyano (C≡N) group bonded to the α-carbon; indices 5–6 are the hydrogen atoms bonded to the β-carbon; indices 7–9 are the amino (NH_2) group; indices 10–13 are the methyl (CH_3) group; and index 14 is the sulfur.

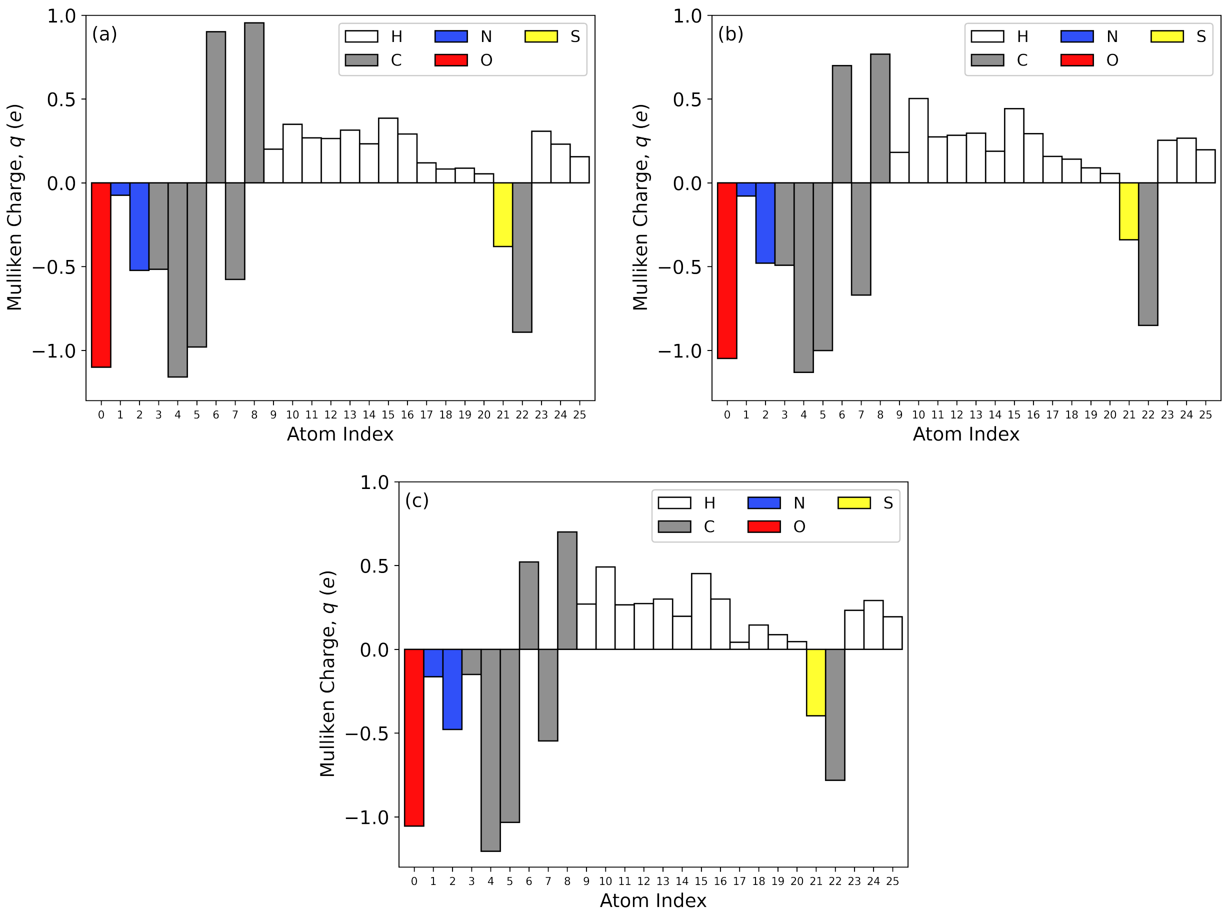

Figure S13. Mulliken charge distributions in the three enolate rotamers after the addition of methanethiolate to 4-(dimethylamino)-2-butenamide. Shown are (a) $\Theta_{\mathrm{min}} = 50.6^\circ$, (b) $\Theta_{\mathrm{min}} = 168.5^\circ$, and (c) $\Theta_{\mathrm{min}} = 307.6^\circ$. Here, indices 0 and 8 are the carbonyl (C=O) group; indices 1, 4, 5, and 11–16 are the dimethylamino [$N(CH_3)_2$] group; indices 2, 19, and 20 are the amino (NH_2) group; indices 6 and 7 are the β- and α-carbons, respectively; indices 3, 9, and 10 are the methylene (CH_2) group (in between the β-carbon and the dimethylamino group); indices 17 and 18 are the hydrogen atoms bonded to the β- and α-carbons, respectively; index 21 is the sulfur; and indices 22–25 are the methyl (CH_3) group.

5 Non-Covalent Interaction Plots

Figures S14 and S15 show plots of the reduced density gradient, $s(\rho)$, versus the electron density multiplied by the sign of the second Hessian eigenvalue, $\mathrm{sgn}(\lambda_2)\rho$, for the various enolate rotamers.[S5] The relationship between ρ and $s(\rho)$ can be seen to be of the form $a\rho^{-1/3}$, for some constant $a \in \mathbb{R}$, which is because atomic and molecular densities are piecewise exponential.[S5] In all cases, points in the low-density, high-gradient region correspond to the exponentially decaying tail regions of the density, far from the nuclei.[S5] In contrast, points in the high-density, low-gradient region correspond to covalent bonds, which have characteristic saddle points in the electron density;[S5] these can be seen at $(\mathrm{sgn}(\lambda_2)\rho, s(\rho)) \approx (-0.2, 0)$ and $(-0.3, 0)$.

The non-covalent interactions can be seen by the spikes in the low-density, low-gradient region. These interactions are mostly bound by $\rho < 0.03\,\mathrm{a.u.}$; this bound was therefore chosen for the visualization of non-covalent interactions.[S6]

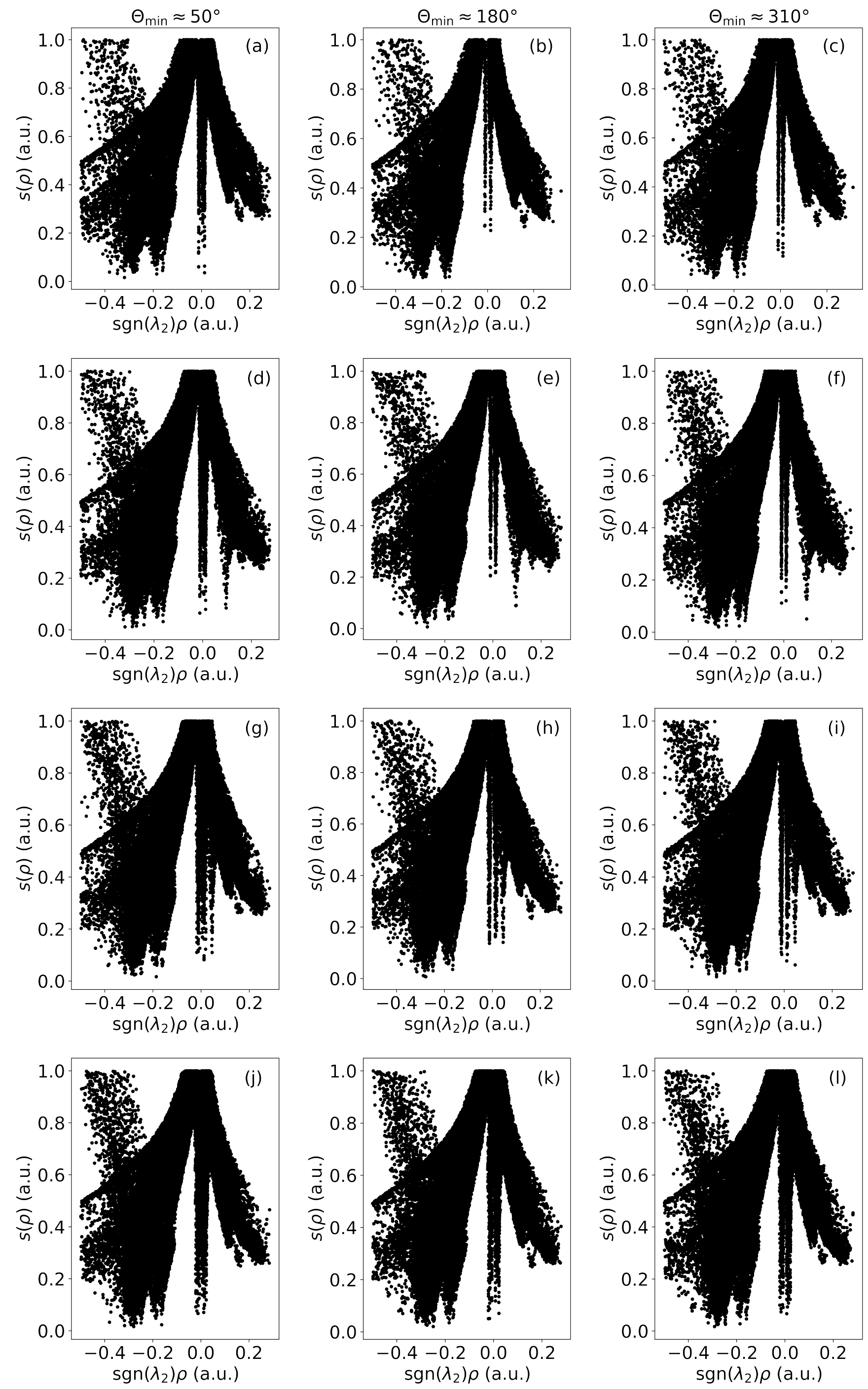

Figure S14. Non-covalent interaction plots for enolate rotamers, with $\Theta_{\mathrm{min}} \approx 50^{\circ}$ (first column), 180° (second column) and 310° (third column), after the addition of methanethiolate to (a)–(c) acrylamide, (d)–(f) azetidinyl vinyl ketone, (g)–(i) pyrrolidinyl vinyl ketone, and (j)–(l) piperidinyl vinyl ketone.

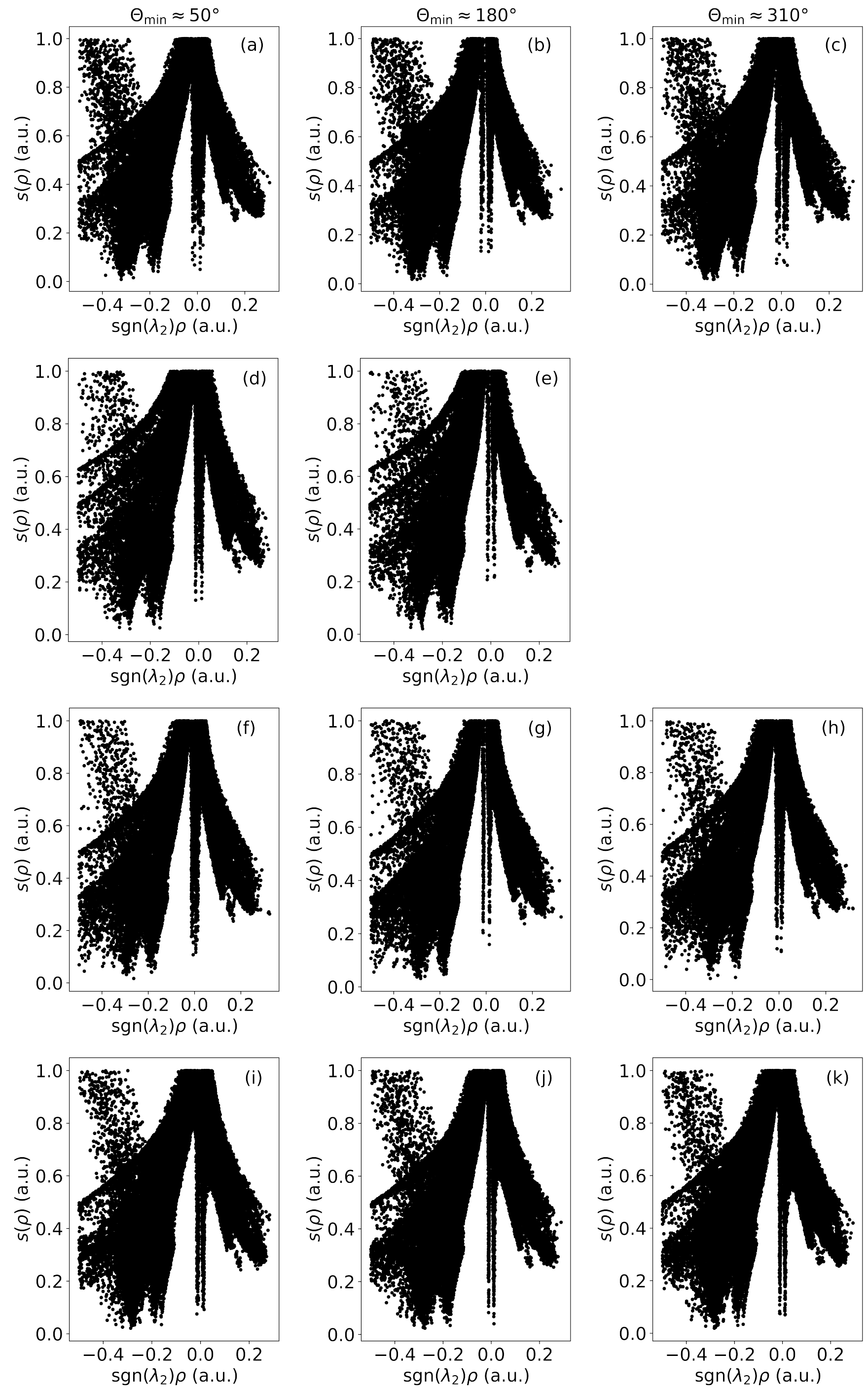

Figure S15. Non-covalent interaction plots for enolate rotamers, with $\Theta_{min} \approx 50^\circ$ (first column), 180° (second column) and 310° (third column), after the addition of methanethiolate to (a)–(c) *N*-phenylacrylamide, (d)–(e) α-fluoroacrylamide, (f)–(h) α-cyanoacrylamide, and (i)–(k) 4-(dimethylamino)-2-butenamide. Note that no third minimum, with $\Theta_{min} \approx 310^\circ$, was found for the nucleophilic addition of methanethiolate to α-fluoroacrylamide.

References

(S1) Lee, T. J.; Rice, J. E.; Scuseria, G. E.; Schaefer III, H. F. Theoretical investigations of molecules composed only of fluorine, oxygen and nitrogen: determination of the equilibrium structures of FOOF, $(NO)_2$ and FNNF and the transition state structure for FNNF *cis-trans* isomerization. *Theor. Chim. Acta* **1989**, *75*, 81–98.

(S2) Mulliken, R. S. Electronic Population Analysis on LCAO–MO Molecular Wave Functions. I. *J. Chem. Phys.* **1955**, *23*, 1833–1840.

(S3) Raghavachari, K.; Trucks, G. W.; Pople, J. A.; Head-Gordon, M. A fifth-order perturbation comparison of electron correlation theories. *Chem. Phys. Lett.* **1989**, *157*, 479–483.

(S4) Lee, T. J.; Taylor, P. R. A Diagnostic for Determining the Quality of Single-Reference Electron Correlation Methods. *Int. J. Quantum Chem.* **1989**, *36*, 199–207.

(S5) Johnson, E. R.; Keinan, S.; Mori-Sánchez, P.; Contreras-García, J.; Cohen, A. J.; Yang, W. Revealing Noncovalent Interactions. *J. Am. Chem. Soc.* **2010**, *132*, 6498–6506.

(S6) Contreras-García, J.; Johnson, E. R.; Keinan, S.; Chaudret, R.; Piquemal, J.-P.; Beratan, D. N.; Yang, W. NCIPLOT: A Program for Plotting Noncovalent Interaction Regions. *J. Chem. Theory Comput.* **2011**, *7*, 625–632.
